# Superfood potential of *Chlorella vulgaris*: productivity and antioxidant boost under simulated moon and microgravity conditions

**DOI:** 10.1038/s41526-025-00550-4

**Published:** 2025-12-12

**Authors:** Giacomo Fais, Filippo Ghiani, Debora Dessì, Mattia Casula, Giovanni Perra, Eleonora Torchia, Nicola Lai, Giacomo Cao, Alessandro Concas

**Affiliations:** 1 Interdepartmental Centre of Environmental Science and Engineering (CINSA), University of Cagliari, Cagliari, Italy; 2 Department of Mechanical, Chemical and Materials Engineering, University of Cagliari, Cagliari, Italy; 3 Department of Life and Environmental Sciences, University of Cagliari, Cagliari, Italy; 4 Center for Advanced Studies, Research and Development in Sardinia (CRS4), Pula, Italy

**Keywords:** Biotechnology, Cell biology, Plant sciences, Health care, Engineering, Agriculture

## Abstract

As space missions extend to the Moon and beyond, Bioregenerative Life Support Systems (BLSS) are vital for food, oxygen, and resource recycling in closed habitats. We examined the physiological, biochemical, and lipidomic responses of *Chlorella vulgaris* (CCALA 269) grown under simulated Earth gravity (1 g), Moon gravity (0.17 g), and microgravity (μg) using a 3D clinostat. Reduced gravity was associated with higher biomass, photosynthetic pigments, and antioxidant capacity. Cultures under lunar and microgravity showed up to 170% more chlorophyll and carotenoids, and 160% more polyphenols and antioxidant activity. Lipidomics revealed membrane remodeling, with higher galactolipids and triacylglycerols, suggesting adaptations to preserve membrane function and energy reserves. These responses indicate substantial physiological plasticity in *C. vulgaris*, suggesting its potential relevance for BLSS as a source of nutrient-rich biomass, oxygen, and antioxidants. Our results suggest its potential for space food and life support, and the need for further research under real partial gravity conditions.

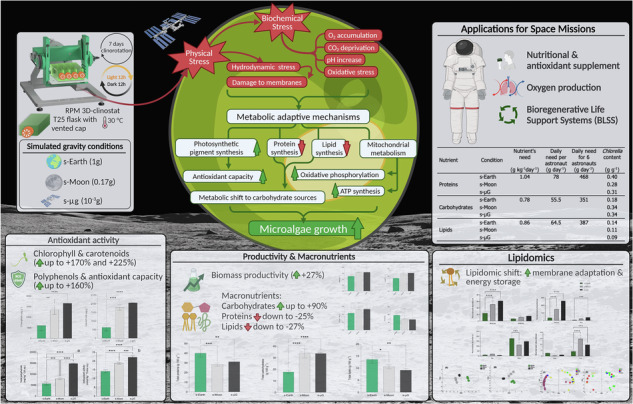

## Introduction

Space exploration represents one of the most ambitious scientific and technological endeavors of the 21st century, requiring innovative solutions to ensure human survival in extreme and inhospitable environments. With the concrete prospect of long-term missions to the Moon, a celestial body of paramount interest for human colonization, it becomes crucial to address the challenges posed by these destinations, including ionizing radiation, altered gravity, and the limited availability of local resources^1^.

The Moon, due to its proximity to Earth and its potential as a testing ground for space exploration technologies, represents a fundamental step toward sustainable human spaceflight. Lunar settlements will allow for the validation of life support systems under reduced gravity conditions (0.17 g), which profoundly affect biological and physical processes, such as fluid dynamics, nutrient transport, and photosynthetic efficiency^2–4^.

Similarly, microgravity, as experienced in orbit aboard space stations or during interplanetary travel, significantly alters cellular metabolism and growth dynamics. At the cellular level, reduced gravity modifies key physical processes, such as gas exchange, sedimentation, and intracellular organization. These changes affect cytoskeletal dynamics, vesicle transport, and membrane tension, modulating mechanosensitive pathways and ultimately cellular metabolism. Both real and simulated microgravity studies have demonstrated that the randomization of the gravity vector activates Ca^2+^-dependent signaling and cytoskeletal remodeling in microorganisms, confirming the functional equivalence of clinostat-based analogs with orbital conditions^5–9^.

Understanding how microalgae respond to these gravitational conditions is key to optimizing their role in Bioregenerative Life Support Systems (BLSS)^10–15^.

While fluctuations in temperature and the absence of atmosphere can be mitigated through advanced thermal isolation, radiation shielding, and pressurized habitats, reduced gravity remains one of the most significant biological and technical challenges^16–19^.

Consequently, establishing stable lunar colonies is essential for enabling long-duration space exploration missions. NASA’s Artemis Program is crucial, with Artemis III planned for 2026 to land astronauts on the lunar surface^20^. The Moon provides a critical platform for advancing autonomous life support systems and resource utilization technologies, while also allowing researchers to refine strategies under altered gravity conditions that could have significant implications for human physiology and biological systems^21,22^.

Sophisticated life support systems (LSS) onboard the ISS already recycle up to 90% of water and partially recover oxygen from exhaled CO_2_^23^. However, their dependency on terrestrial resupply missions for food renders them unsuitable for long-term missions beyond low Earth orbit (ESA Annual Report^24^). Additionally, research conducted in the microgravity environment of the ISS has provided invaluable insights into the physiological and biochemical responses of biological systems in the absence of gravity-driven convection and sedimentation processes^21^.

For lunar and long-duration spaceflight, the development of BLSS is essential. These systems integrate biological and physicochemical processes to create closed, self-sustaining ecosystems capable of providing astronauts with oxygen, water, and food while recycling organic waste^25,26^.

Among the biological components suitable for BLSS, microalgae stand out for their exceptional versatility, combining high photosynthetic efficiency, rapid growth rates, and resilience under extreme conditions with minimal resource inputs. Their capacity to produce both edible biomass and oxygen makes them particularly promising for space applications^11,27–29^. Among the most studied microalgae, *Chlorella vulgaris* spp. has emerged as a leading candidate for BLSS due to its ability to accumulate biomass rich in nutrients and bioactive compounds^30–34^. On Earth, it is widely utilized in food products and dietary supplements, and it is officially classified as GRAS (Generally Recognized as Safe)^10,35^. Moreover, its secondary metabolites, such as carotenoids and polyphenols, provide antioxidant properties crucial for mitigating oxidative stress, a significant challenge during prolonged space missions characterized by high levels of ionizing radiation^36–38^.

Previous experiments conducted by our group explored the cultivation of *C. vulgaris* under conditions simulating synthetic urine as nutrient sources^39^. These studies demonstrated its ability to grow efficiently while maintaining a rich nutritional profile. Remarkably, biomass produced in regolith-based media exhibited elevated protein and carbohydrate levels, underscoring its potential as a sustainable food source. These findings align with In-situ Resource Utilization (ISRU) principles, highlighting the role of local resources in reducing terrestrial supply dependency^39^. Moreover, *C. vulgaris* displayed resilience to ionizing radiation, maintaining stable growth and metabolic activity while increasing the production of pigments, such as chlorophylls and carotenoids, essential for photosynthesis and astronaut health^39^.

However, cultivating microalgae in extraterrestrial environments involves significant challenges, particularly under conditions of altered gravity. Research onboard the ISS has revealed that microgravity impacts photosynthetic metabolism, fluid dynamics, and intracellular metabolite distribution. For instance, recent studies showed that *Limnospira indica* suffered reduced photosynthetic efficiency and growth in microgravity due to increased oxygen saturation and changes in fluid boundary layers^40^. Conversely, *Euglena gracilis* exhibited remarkable adaptability under microgravity, effectively contributing to CO_2_ removal and oxygen production^41,42^.

Studies on *C. vulgaris* under microgravity have shown significant cellular adaptations, including enhanced mitochondrial activity and structural changes that sustain metabolic function^43^. However, oxidative stress induced by microgravity remains a critical concern. For example, other microalgae species, such as *Anabaena sp*. and *Synechococcus 7942*, showed increased lipid peroxidation and membrane instability under simulated microgravity conditions^44,45^. These findings highlight the intricate relationships between gravity and physiological processes, underscoring the need for additional research to optimize LSS for extended-duration missions.

To address these challenges, this study evaluates *C. vulgaris* across varying gravitational conditions, microgravity (μg), lunar gravity (0.17 g), and terrestrial gravity (1 g), to understand how different levels of gravitational stress affect its growth, productivity, and chemical composition. This study focuses on critical parameters, including biomass composition and the production of bioactive compounds, such as carotenoids and polyphenols. The findings will aid in optimizing BLSS for lunar bases and space habitats, serving as a cornerstone for future advancements in closed-loop space food production.

## Results and discussions

### Effect of simulated earth, moon, and microgravity conditions on biomass productivity and growth kinetics in *Chlorella vulgaris*

**Fig. 1:**
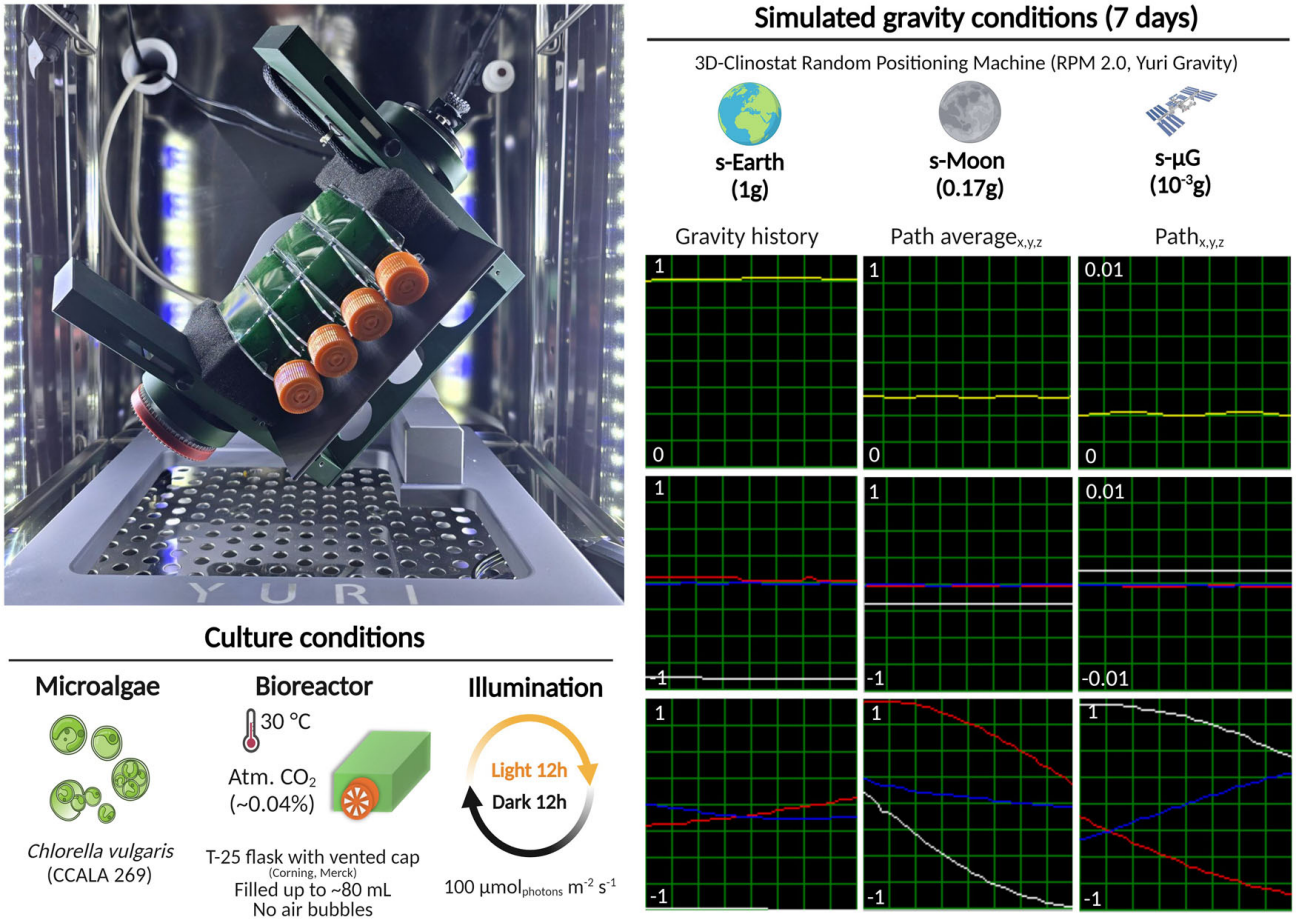
Experimental setup used for the investigation of the effect of simulated gravity conditions, Earth (s-Earth), Moon (s-Moon), and microgravity (s-μg), on *Chlorella vulgaris* by using a Random Positioning Machine (RPM) 3D-clinostat (RPM 2.0, Yuri Gravity). Microalgae strain, bioreactor type, and illumination system specifications are reported in culture conditions section. The clinorotation time that was tested is reported in simulated gravity conditions section together with the average and path average (x, y, and z) accelerations that derive from the software of RPM 2.0 (Yuri Gravity). Created with BioRender.com.

The cultures of *C. vulgaris* CCALA 269 were cultivated under simulated s-Earth, s-Moon, and s-μg conditions, starting from an initial optical density (OD) of approximately 0.2 at 650 nm and a uniform pH, which was monitored daily throughout the experiment. During the early growth phase (days 0–3), s-Earth exhibited a slightly higher biomass accumulation compared to s-Moon and s-μg. However, after day 4, the growth rate in s-Earth slowed, leading to a lower final biomass concentration (1.25 ± 0.11 g L^−1^), while both s-Moon and s-μg cultures maintained a steady growth, reaching plateau values of 1.59 ± 0.02 g L^−1^ and 1.59 ± 0.05 g L^−1^, respectively, corresponding to an approximately 27.2% higher biomass yield compared to s-Earth (Fig. 1).

This pattern suggests that while Earth gravity may initially support faster growth, the observed biomass plateau during prolonged cultivation could be influenced by factors, such as nutrient depletion, metabolite accumulation, or inefficient gas exchange. In contrast, s-Moon and s-μg conditions supported continuous biomass production, potentially due to reduced sedimentation and improved resource distribution^46^. These findings align with results from the PBR@LSR experiment conducted on the ISS, where in situ microgravity was achieved under true weightlessness conditions using a continuously aerated photobioreactor with active gas-exchange control^47^. Although initial growth was faster in the Earth-based control, low sedimentation effects may have caused spatial heterogeneity, restricting nutrient and light availability for some cells and thereby potentially constraining overall productivity^47^. Conversely, in s-Moon and s-μg, the sedimentation was even lower compared to s-Earth and ensured uniform cell dispersion, optimizing resource accessibility and sustaining metabolic activity over time^47^.

Similarly, the higher biomass productivity observed in s-Moon and s-μg cultures further supports the hypothesis that reduced gravity environments mitigate growth constraints associated with cell aggregation and nutrient transport limitations^18^. Moreover, our findings are consistent with previous research on *Arthrospira platensis* (Spirulina), where a slight productivity increase was observed under microgravity simulated using a similar Random Positioning Machine (RPM), where cultivation was performed in vented-cap T-25 flasks filled to capacity compared to the same cultures under 1 g static conditions^18^. This is further reinforced by bacterial studies aboard the ISS, which have shown that real microgravity does not negatively impact final cell concentrations, confirming that microbial bioproduction systems can function effectively both in space and on Earth^48^.

**Fig. 2:**
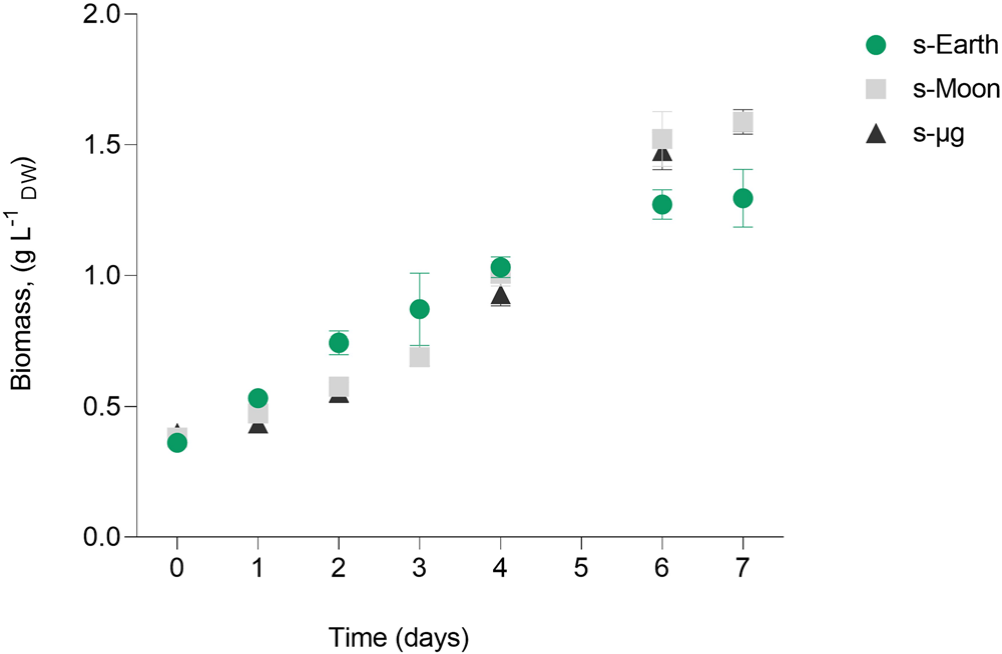
Effect of simulated Earth (s-Earth), Moon (s-Moon), and microgravity (s-μg) conditions on biomass production of *Chlorella vulgari*s. Biomass concentration (g L^−1^, dry weight) was monitored over 7 days to evaluate the effect of different gravity conditions on microalgal growth. Data are obtained using the calibration curve (Supplementary Fig. 1) and are presented as mean ± standard deviation (SD) (*n* = 3).

However, responses from *Chlorella* spp. to simulated microgravity have shown variability depending on the experimental setup. Mills and Pierson^19^ reported significantly reduced growth under simulated microgravity, with biomass reaching only 42% of that obtained under normal gravity after 14 days. Despite this, the photosynthetic capacity of the cells remained unaffected, suggesting that microgravity primarily influences cellular growth and division rather than photosynthetic efficiency. The discrepancy with our findings may stem from differences in experimental conditions, particularly the use of a 3D clinostat (Random Positioning Machine) (Fig. 1) in our study versus the HARV bioreactor used by Mills and Pierson^19^. The clinostat provides a low-shear environment, which may better support *C. vulgaris* growth compared to the HARV system, where fluid dynamics could impose additional mechanical stress on the cells^19,49^ (Fig. 2).

**Fig. 3:**
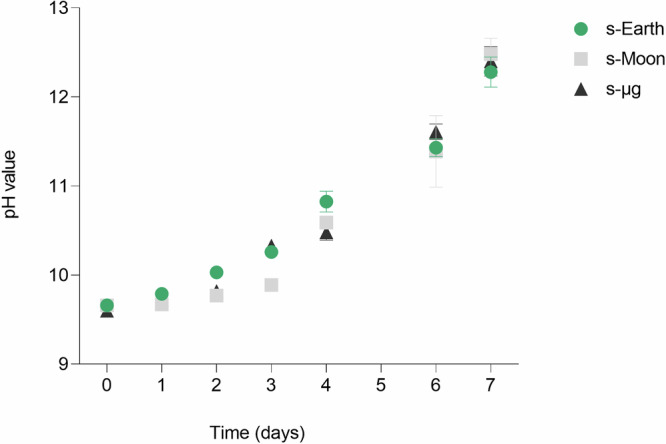
pH dynamics in the culture medium during the growth of *Chlorella vulgaris* under simulated Earth (s-Earth), Moon (s-Moon), and microgravity (s-μg) conditions. The pH of the culture medium was monitored daily for 7 days to evaluate changes associated with microalgal growth and metabolic activity under different gravity simulations. Data are shown as mean ± SD (*n* = 3).

Further comparison can be drawn with the study by Ellena et al.^40^ on *Limnospira indica*, where microgravity was simulated with an RPM and using gas-permeable culture bag systems led to reduced photosynthetic efficiency and growth, mainly due to oxygen saturation and increased fluid boundary layer thickness. In contrast, *C. vulgaris* in our experiment did not exhibit such limitations. The stability of pH dynamics across s-Moon and s-μg conditions suggests sustained photosynthetic activity, as the gradual pH increase indicates continuous CO_2_ consumption and carbonic acid equilibrium shifts. Although direct measurements of photosynthetic efficiency were not performed, the absence of abrupt pH fluctuations (Fig. 3) or stagnation supports the hypothesis that *C. vulgaris* maintained active carbon assimilation and metabolic function under these conditions.

Additionally, during growth under s-Earth, s-Moon, and s-μg conditions, all cultures started with a similar initial pH ( ~ 10) and exhibited a steady increase, reaching a plateau at approximately 12, regardless of gravitational condition.

In our experiment, the cultures were conducted in airtight bioreactors with gas-permeable caps, following a standardized protocol for clinostat-based experiments^50,51^. This approach is essential to prevent bubble formation and minimize mechanical shear stress, ensuring a stable and controlled environment that mimics microgravity conditions. While optimal for reducing mechanical artifacts, this configuration significantly limits active CO_2_ exchange with the external environment, as the gas exchange surface is restricted to the small area of the filter embedded in the cap. Consequently, because gas transfer through the small diffusion area of the vented cap occurs primarily near the gas-liquid interface, CO_2_ replenishment is spatially limited. This configuration may create diffusion-controlled microgradients of dissolved inorganic carbon, leading to locally reduced CO_2_ availability in regions farther from the interface, a well-documented phenomenon in semi-closed algal cultures^9,52^.

**Fig. 4:**
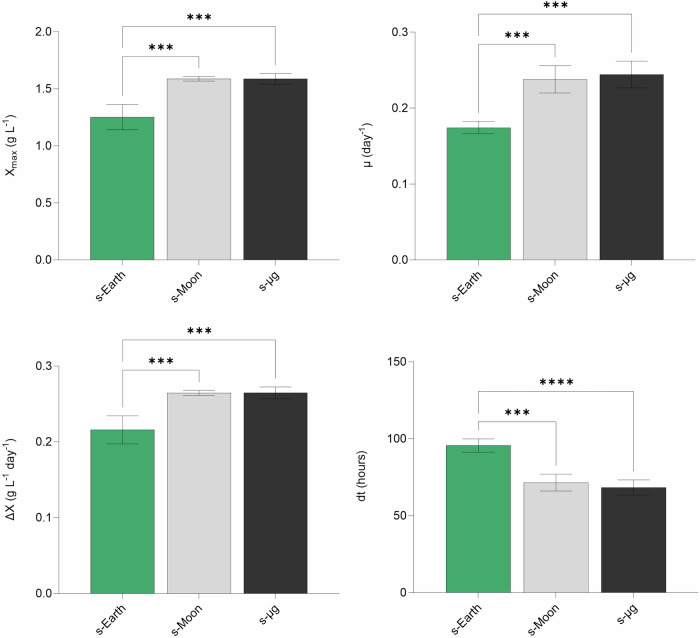
Growth parameters of *Chlorella vulgaris* under simulated Earth (s-Earth), Moon (s-Moon), and microgravity (s-μg) conditions. The graphs display key growth metrics, including maximum biomass concentration (*X*_*max*_, g L^−1^), specific growth rate (*μ*, day^−1^), biomass productivity (Δ*X*, g L^−1^ day^−1^), and doubling time (*dt*, hours). Bars indicate mean ± SD (*n* = 3). Statistical significance is indicated by asterisks (***p < 0.001, ****p < 0.0001), showing significant differences between conditions.

Despite all culture conditions showing a similar pH trend, reaching a plateau around 12, biomass productivity and composition differed significantly among conditions (Fig. 4). Specifically, cultures under s-Moon and s-μg conditions achieved higher biomass concentrations (1.59 ± 0.02 g L^−1^ and 1.59 ± 0.05 g L^−1^, respectively) compared to Earth gravity (1.25 ± 0.11 g L^−1^). This discrepancy indicates that, while pH serves as an indicator of metabolic balance, it is not the sole determinant of final productivity. The reduced gravity environment likely facilitated a more homogeneous nutrient distribution and improved photosynthetic efficiency, allowing microalgae to fully exploit available resources until CO_2_ depletion.

These findings suggest that while pH stability is crucial for maintaining cellular homeostasis, additional mechanisms influence *C. vulgaris* growth under reduced gravity conditions. Previous studies have shown that cellular adaptations of *Chlorella* cells exposed to microgravity conditions both aboard orbital spacecraft (13-day biosatellite Bion-9/-10, spaceflight, 12-month cultivation in orbital station Mir) and in slow-rotating horizontal clinostat 35-day simulations, particularly enhanced mitochondrial activity and ultrastructural rearrangements, may contribute to sustained metabolic function in altered gravity environments. While structural modifications in chloroplasts have also been reported^53–56^, their direct role in maintaining photosynthetic efficiency under microgravity remains to be fully elucidated^43^.

Furthermore, regarding kinetic growth parameters, the specific growth rate (*μ*) was significantly higher under s-Moon and s-μg conditions (0.24 ± 0.02 day^−1^) compared to Earth gravity (0.17 ± 0.01 day^−1^), indicating that reduced gravity enhances the replication capacity of *C. vulgaris*. This improvement in growth kinetics is likely linked to a more uniform nutrient distribution, facilitated by reduced sedimentation and cell aggregation. The resulting homogeneous microalgal suspension ensures that cells have consistent access to both light and dissolved nutrients, thereby enhancing overall metabolic activity.

The average biomass productivity (Δ*X*) also reflected this trend, with cultures under s-Moon and s-µg conditions achieving 0.26 ± 0.00 g L^−1^ day^−1^ and 0.26 ± 0.01 g L^−1^ day^−1^, respectively, compared to 0.22 ± 0.02 g L^−1^ day^−1^ under Earth gravity. This enhanced productivity may be attributed to the ability of *C. vulgaris* to maintain high photosynthetic efficiency even as dissolved CO_2_ levels diminished. The limited gas exchange in the bioreactor setup likely imposed a natural constraint on CO_2_ availability, yet the microgravity environment appears to have mitigated this limitation by promoting a more efficient utilization of the available carbon resources. A crucial parameter that further supports the positive impact of reduced gravity is the doubling time (*dt*). Cultures under s-Moon and s-μg conditions showed significantly shorter doubling times (71.52 ± 5.49 h and 68.38 ± 4.91 h, respectively) compared to s-Earth (95.56 ± 4.36 h). Shorter doubling times reflect not only accelerated growth rates but also increased efficiency in cellular processes, from nutrient uptake to biomass conversion. This parameter is particularly important for space missions, where maximizing biomass production in a limited period is critical for sustaining life support systems (LSS).

### Photosynthetic pigments and antioxidant activity under simulated gravity conditions

**Fig. 5:**
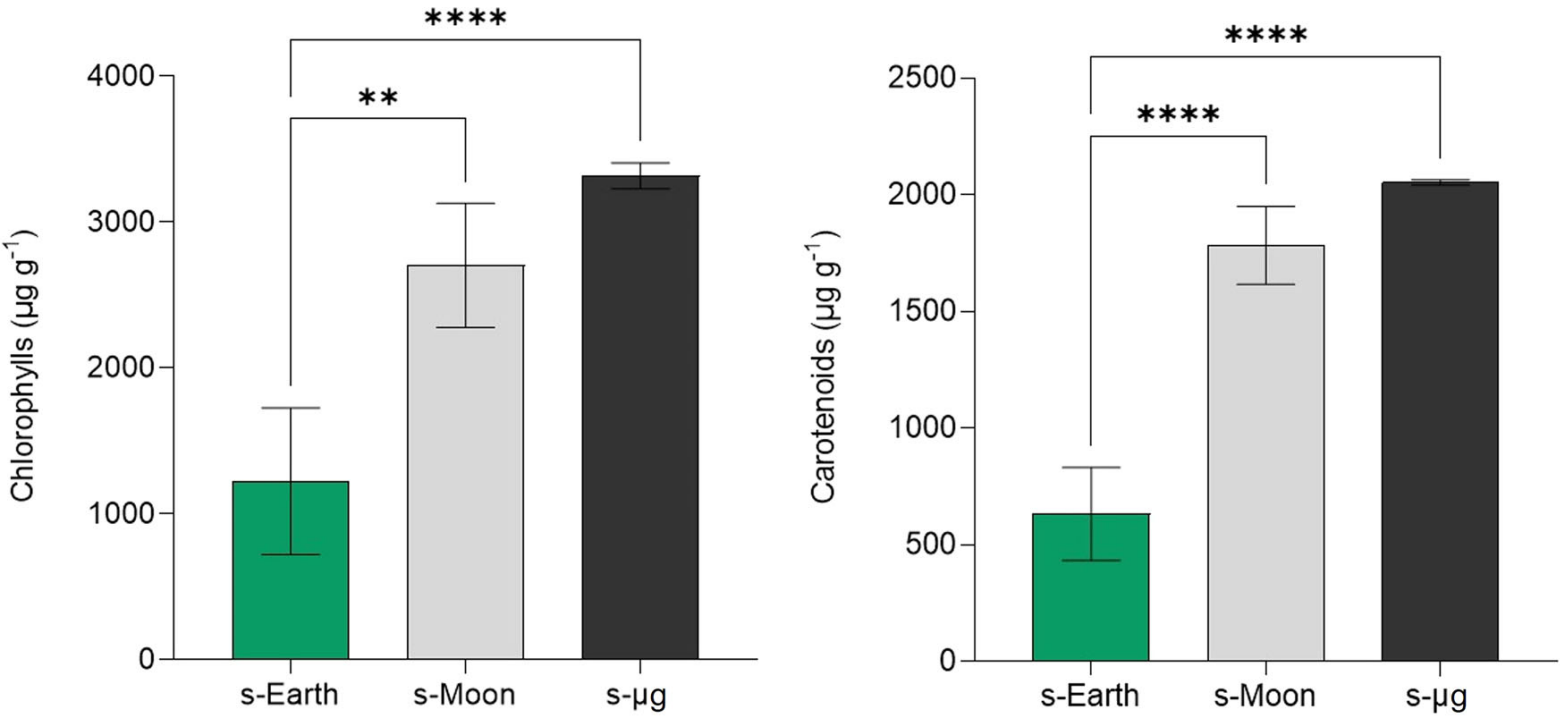
Chlorophyll and carotenoid content in *Chlorella vulgaris* cultivated under simulated Earth (s-Earth), Moon (s-Moon), and microgravity (s-μg) conditions. Bars indicate mean ± SD (*n* = 3). Significant differences in the synthesis of photosynthetic pigments across gravity conditions are denoted by asterisks (*p < 0.05, **p < 0.01, ***p < 0.001, ** ** *p* < 0.0001).

The enhanced growth performance of *C. vulgaris* under reduced gravity conditions can be attributed to specific cellular adaptations that optimize energy production and improve the efficiency of the photosynthetic apparatus. Beyond biomass accumulation, these adaptations involve the synthesis of photosynthetic pigments, such as chlorophylls and carotenoids, as well as antioxidant compounds, including polyphenols, which play a crucial role in cellular protection against oxidative stress^57^. In particular, polyphenols, along with carotenoids, represent essential defense molecules that contribute to maintaining cellular homeostasis under the oxidative pressure imposed by microgravity and lunar gravity environments^58^. Given the potential stressors associated with microgravity and lunar gravity, such as altered gas exchange, oxidative imbalances, and variations in nutrient availability, the accumulation of these molecules provides valuable insights into the physiological responses of *C. vulgaris* to different gravitational environments, with significant implications for potential biomass applications, particularly in the field of space nutrition^59^. Chlorophylls are essential for light harvesting and energy conversion in photosynthesis, while carotenoids function both as accessory pigments and as antioxidants, mitigating the effects of reactive oxygen species (ROS). However, when included in the diet, carotenoids also play a fundamental role in human health, acting as potent antioxidants capable of neutralizing free radicals and reducing the risk of degenerative diseases^60^. Furthermore, certain carotenoids, such as β-carotene, can be converted into vitamin A, which is essential for vision, immune function, and cellular health^61,62^. Therefore, the enrichment of *C. vulgaris* biomass with these bioactive compounds could enhance its nutritional value, making it an even more promising resource for food and nutraceutical applications, particularly in space exploration and extreme environments where a balanced diet is crucial for human well-being. These physiological adaptations are reflected in the pigment composition of *C. vulgaris*, as indicated by the significant increase in chlorophyll and carotenoid content under reduced gravity conditions. Our analysis revealed that chlorophyll and carotenoid content were significantly higher under s-Moon and s-μg conditions compared to s-Earth, suggesting that reduced gravity environments promote enhanced photosynthetic capacity and light absorption efficiency. Specifically, chlorophyll concentration increased from 1223.46 ± 502.52 μg g^−1^ in s-Earth to 2703.57 ± 425.16 μg g^−1^ in s-Moon and 3316.82 ± 89.08 μg g^−1^ in s-μg. Similarly, carotenoid levels followed the same trend, rising from 631.80 ± 199.62 μg g^−1^ in s-Earth to 1783.00 ± 167.10 μg g^−1^ in s-Moon and 2052.76 ± 11.76 μg g^−1^ in s-μg (Fig. 5).

**Fig. 6:**
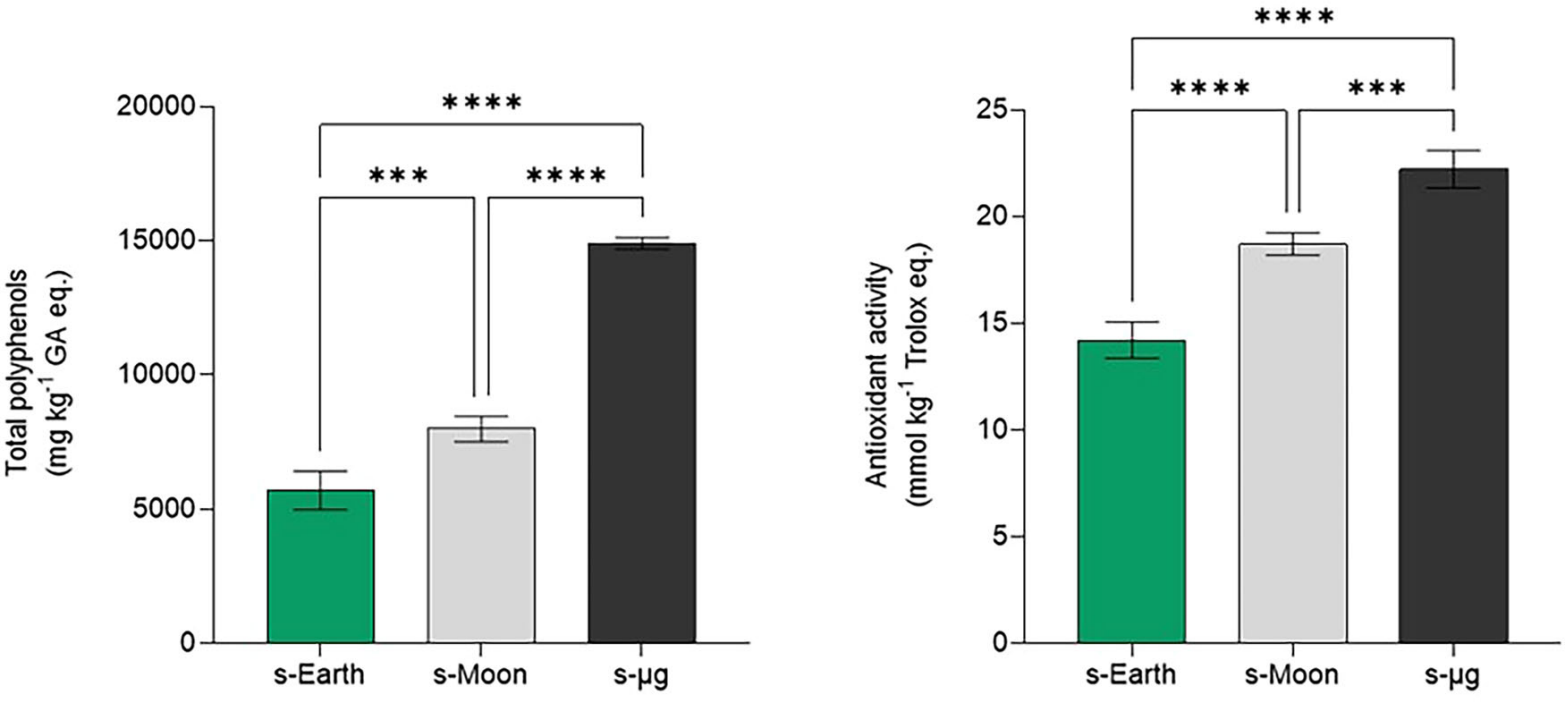
Total polyphenols and Antioxidant activity in *Chlorella vulgaris* cultivated under simulated Earth (s-Earth), Moon (s-Moon), and microgravity (s-μg) conditions. Bars indicate mean ± SD (*n* = 3). Significant differences in chlorophyll synthesis across gravity conditions are denoted by asterisks (*p < 0.05, **p < 0.01, ***p < 0.001, ** * * *p* < 0.0001).

These changes correspond to a 121% increase in chlorophyll content under s-Moon conditions and a 171% increase under s-μg conditions compared to s-Earth. Interestingly, similar trends have been observed in other photosynthetic microorganisms exposed to microgravity. For instance, *Synechocystis sp*. PCC 6803 showed enhanced synthesis of photosynthetic pigments, including components of the phycobilisome complex, when cultivated under microgravity conditions simulated on a 1D clinostat for 5 days compared to 1 g static control cultures^63^. Although no significant difference in growth was recorded, the marked increase in pigments indicates a common adaptive strategy among cyanobacteria and microalgae to maintain photosynthetic efficiency under gravitational stress. (Fig. 6).

Similarly, carotenoid levels exhibited an even more pronounced rise, with an 182% increase in s-Moon and a 225% increase in s-μg, indicating a consistent influence of reduced gravity on photosynthetic pigment accumulation. In this perspective, the carotenoid/chlorophyll (Car/Chl) ratio was also calculated to further characterize the response of *C. Vulgaris* to altered oxidative conditions under reduced gravity. The Car/Chl ratio slightly increases from 0.53 ± 0.06 in s-Earth to 0.66 ± 0.05 in s-Moon and 0.62 ± 0.01 in s-μg, potentially suggesting a gravity-dependent adaptation of the antioxidant pigments (Supplementary Table 2). This trend suggests that *C. Vulgaris* may establish a new equilibrium between carotenoids and chlorophylls as part of an adaptive response to the altered environmental conditions^64,65^. The selective and significant increase in the Car/Chl ratio at s-Moon compared to s-Earth may reflect a hormetic-type response, in which a moderate stress triggers an adaptation of photoprotective pigment synthesis without disrupting the overall photosynthetic pigment equilibrium^66^. In addition, these results indicate that *C. vulgaris* may upregulate pigment biosynthesis under reduced gravity conditions, potentially as a compensatory strategy to optimize photon capture and maintain efficient photosynthesis despite environmental challenges^67–69^. Additionally, the significant increase in carotenoids under s-Moon and s-μg conditions suggests an enhanced protective response against oxidative stress, supporting the hypothesis that microgravity induces a cellular stress response that favors antioxidant accumulation^67,69–71^.

In this context, our data also reveals a dramatic increase in polyphenol content under reduced gravity, reinforcing the role of *C. vulgaris* as a natural antioxidant source. Total polyphenols rose from 5688 ± 720 mg kg^−1^ GAE in s-Earth to 7980 ± 470 mg kg^−1^ in s-Moon, reaching 14885 ± 216 mg kg^−1^ in s-μg, thus more than doubling under microgravity. This increase was mirrored by a rise in total antioxidant activity, indicating that polyphenol biosynthesis may act as a targeted defense mechanism to counteract oxidative imbalance in space-relevant conditions.

These findings indicate the polyphenols, together with chlorophylls and carotenoids, are important molecules in the adaptation of *C. vulgaris* to reduced gravity. Regarding the modest differences observed between s-Moon and s-μg conditions, we hypothesize the involvement of a threshold-based mechanism. Although the gravitational load differs only slightly in numerical terms (0.17 g vs. 0.001 g), both levels are substantially lower than s-Earth gravity and may cross a common activation threshold for antioxidant responses. It is therefore plausible that exposure to s-Moon is already sufficient to trigger key protective pathways, and that further reduction to s-μg induces only limited additional activation of these systems. The strong increase in polyphenols and antioxidant activity suggests that the cells enhance their defense systems to protect against oxidative stress. These results confirm the relevance of such compounds for space biotechnologies and astronaut nutrition^58,72^. Overall, the metabolic changes observed indicate that *C. vulgaris* adjusts its physiology under reduced gravity to better cope with oxidative challenges. The combined rise in polyphenols, chlorophylls, carotenoids, and total antioxidant capacity suggests a coordinated response to improve cellular protection and maintain function in space-like environments. These antioxidant-rich profiles could enhance the functional food potential of *C. vulgaris* biomass in space diets, offering both essential nutrients and bioactive molecules to counteract oxidative stress and support astronaut health. This biochemical profile under s-Moon and s-μg conditions suggests that *C. vulgaris* could serve as a promising model for BLSS in space. Its high antioxidant and pigment production makes it a valuable nutritional resource for astronauts, offering protection against space-induced oxidative stress while supporting overall health^73,74^. These adaptations improve its functional food potential and reinforce its feasibility as a sustainable, in situ food source for long-duration missions, reducing reliance on Earth-based supplies and enhancing dietary diversity in space.

### Macronutrient composition of *Chlorella vulgaris* under simulated earth, moon, and microgravity conditions

**Fig. 7:**
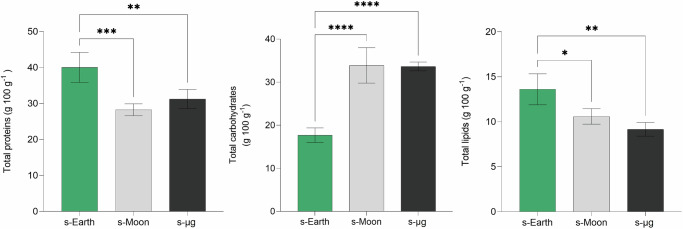
Total proteins, carbohydrates, and lipids in *Chlorella vulgaris* cultivated under simulated Earth (s-Earth), Moon (s-Moon), and microgravity (s-μg) conditions. **Bars indicate mean** **±** **SD (*n*** = **3)**. Significant differences across gravity conditions are denoted by asterisks (*p < 0.05, **p < 0.01, ***p < 0.001, ** ** *p* < 0.0001).

The metabolic response of *Chlorella vulgaris* to reduced gravity conditions leads to substantial changes also in macronutrient composition, reflecting adaptive strategies that ensure cellular functionality under environmental stressors (Fig. 7).

Proteins, carbohydrates, and lipids, key components of cellular biomass, serve distinct yet interconnected roles, shaping the nutritional potential and physiological behavior of *C. vulgaris* under reduced gravity. Proteins are essential for enzymatic reactions and metabolic regulation, yet their synthesis is significantly impacted by gravitational stress. Our findings reveal a marked decline in protein under s-Moon and s-μg conditions, with levels dropping by 29.4% and 21.9%, respectively, compared to Earth-based controls. This reduction may suggest a shift in nitrogen metabolism, possibly due to altered enzymatic activity or reduced nutrient assimilation efficiency. This decline may also reflect a metabolic trade-off, wherein algae prioritize carbohydrate accumulation as an alternative energy reserve to overcome environmental stressors^19,75,76^. Indeed, carbohydrates exhibited the opposite trend, nearly doubling in both s-Moon and s-μg conditions relative to s-Earth. This hints at a regulatory adaptation, favoring carbon storage over protein synthesis and lipid metabolism to meet the increased energy demands of altered gravity. Such metabolic shift aligns with the work of Popova^43^, who observed profound mitochondrial adaptations in *C. vulgaris* under similar conditions obtained in a slow-rotating clinostat up to 35 days. For instance, the volume of mitochondria per cell increased significantly, from 2.2% in controls to 5.3% in long-term clinorotation, accompanied by denser mitochondrial matrices and more structured cristae. Popova^43^ also reported enhanced oxidative phosphorylation (OXPHOS) and ATP production due to an increase in the activity of key mitochondrial enzymes, such as succinate dehydrogenase (SDH) and Mg^2+^-ATPase, reinforcing the idea that *C. vulgaris* boosts respiratory activity and metabolism to meet energy demands under gravitational stress.

Alongside these metabolic alterations, lipids, essential for membrane integrity and long-term energy storage, exhibited a marked reduction, decreasing by 22.2% under s-Moon and by 32.7% in s-μg, compared to s-Earth. This pattern suggests a downregulation of lipid biosynthesis, likely as an adaptive response to maintain membrane fluidity under gravitational stress, as cells reorganize their structures to cope with microgravity-induced challenges^44,45,77,78^. The simultaneous decrease in lipid content further supports this hypothesis of metabolic reallocation. Carbohydrates, being more readily accessible and less dependent on oxygen availability than lipids, become the preferred energy source. Instead of storing excess carbon as lipids, *C. vulgaris* appears to channel it toward mitochondrial activity and respiratory efficiency, optimizing its metabolism to ensure growth and survival in space-like environments.

Another plausible explanation for this lipid decline lies in the concurrent increase of antioxidant compounds, such as polyphenols and carotenoids, which could reduce the need for lipid-based oxidative defenses^44,77^.

Notably, these adaptive strategies sharply contrast with those observed in *Limnospira indica* under simulated microgravity, as reported by Ellena et al.^40^. In *L. indica*, microgravity caused growth inhibition, suppression of carbon fixation, and downregulation of nitrogen assimilation, indicating a general metabolic slowdown rather than adaptation. This divergence between *C. vulgaris* and *L. indica* likely results from multiple factors. Firstly, species-specific stress response mechanisms play a crucial role. *C. vulgaris* exhibits a high degree of metabolic flexibility, reallocating resources to sustain mitochondrial function and antioxidant production, while *L. indica* appears more susceptible to oxygen and carbon imbalances, leading to metabolic shutdown. Secondly, differences in experimental conditions, including gas exchange efficiency, medium composition, and light intensity, significantly influence metabolic responses. Popova^43^ had already pointed out that reduced oxygen diffusion in microgravity may favor RuBisCO oxygenation over carboxylation, limiting carbon fixation. Similarly, Ellena et al.^40^ observed the formation of a thicker boundary layer around *L. indica* cells under low-shear conditions, which led to intracellular oxygen accumulation, CO_2_ limitation, and metabolic stress. These effects are highly dependent on the design and aeration of cultivation systems, suggesting that *C. vulgaris*, grown under different conditions, may have avoided similar limitations. Finally, fundamental differences in metabolic prioritization emerge: *L. indica* adopts an energy-conservation strategy, downregulating photosynthesis and biomass production to minimize energy expenditure, while *C. vulgaris* could enhance respiration and antioxidant production, suggesting a proactive metabolic adaptation.

The exclusive doubling of carbohydrate reserves under s-Moon and s-µg, despite identical mixing protocols, potentially reflects gravity-mediated reprogramming of carbon allocation, consistent with up-regulation of starch-biosynthesis enzymes documented under reduced gravity^19,40,75,76^.

These contrasting metabolic strategies are also reflected in the final biochemical composition of the biomass, particularly in the macronutrient balance relevant for astronaut nutrition. Based on the nutritional profiles obtained in this study, *C. vulgaris* biomass produced under s-Earth, s-Moon, and s-μg conditions could serve as a compact, sustainable macronutrient source for astronaut diets.

The caloric value of the biomass remains consistently high across all tested conditions, ranging between 342 and 353 kcal per 100 g of dry biomass (Table 1), with modest variability attributable to gravity-related metabolic adjustments.

**Table 1:** Macronutrient and caloric composition of *Chlorella vulgaris* biomass cultivated under simulated Earth (s-Earth), Moon (s-Moon), and microgravity (s-μg) conditions

Condition	Proteins (g/100 g D.W.)	Carbohydrates (g/100 g D.W.)	Lipids (g/100 g D.W.)	Calories (kcal/100 g D.W.)
s-Earth	40.02 ± 4.16	17.69 ± 1.71	13.61 ± 1.72	353.33 ± 23.73
s-Moon	28.26 ± 1.65	33.90 ± 4.12	10.59 ± 0.86	343.95 ± 19.37
s-μg	31.24 ± 2.67	33.66 ± 1.01	9.16 ± 0.76	342.04 ± 13.31

Values are expressed per 100 g of dry biomass as mean ± SD (*n* = 3). Caloric content was calculated based on protein, carbohydrate, and lipid values following FAO guidelines^81^.

Although protein content is higher under s-Earth, the increased carbohydrate content observed in s-Moon and s-μg compensates for this reduction, yielding a biomass that remains energy-rich and suitable for functional food formulations. Protein levels decrease by approximately 29% in s-Moon and 22% in s-μg relative to s-Earth, while carbohydrates almost double, increasing by 91% and 90%, respectively. Lipid content also declines, with a reduction of 22% in s-Moon and 33% in s-μg. Despite these adjustments, the total caloric value of *C. vulgaris* biomass remains high across gravity conditions, ensuring its potential role as a compact energy source for space missions.

From a quantitative perspective, macronutrient ratios shift substantially with gravity. Under s-Earth, *C. vulgaris* shows a protein-dominant profile with a protein:carbohydrate: lipid ratio of approximately 2.3:1:0.8. In contrast, under s-Moon and s-μg, this ratio evolves toward a more carbohydrate-rich profile, reaching approximately 1:1.2:0.37 (s-Moon) and 1:1.1:0.29 (s-μg), indicating a physiological prioritization of carbohydrate accumulation over proteins and lipids under gravitational stress. This shift suggests that *C. vulgaris* adapts to reduced gravity by reallocating metabolic resources toward carbohydrates as an immediate and flexible energy reserve, likely to sustain high respiratory activity under microgravity, as also proposed by Popova^43^ regarding mitochondrial adaptation. In turn, protein synthesis is reduced, possibly reflecting limitations in nitrogen metabolism or a strategic reduction in energetically costly biosynthetic processes under stress. Interestingly, when compared to the recommended macronutrient composition for astronaut diets, approximately 15% protein, 30% lipids, and 55% carbohydrates on an energy^79^, *C. vulgaris* biomass grown under s-Moon and s-μg conditions presents a highly carbohydrate-enriched profile but a suboptimal lipid content. This suggests that, while *C.Vulgaris* could serve as an excellent protein and carbohydrate source; additional lipid-rich components would be necessary to balance astronaut diets. Nevertheless, the high carbohydrate fraction could be advantageous for quick energy replenishment and counteracting the chronic energy deficit often observed in space missions^79^. Moreover, considering that carbohydrates and proteins together represent more than 60% of the biomass in all conditions, *C. vulgaris* could be formulated into energy-dense food modules capable of supporting high-calorie dietary requirements, while minimizing payload and storage constraints, as emphasized in NASA and ESA guidelines for long-duration missions^80^. According to FAO-based conversion methods^81^, these values would enable the production of compact, high-calorie biomass units, potentially fulfilling a significant portion of astronauts’ dietary intake while minimizing storage volume and system mass^59,82^. Thus, *C. vulgaris* cultivated under space-like conditions could contribute to BLSS as a multifunctional biological component, with potential implications for nutrition, oxygen production, and crew well-being through the intake of fresh biomass, thereby supporting crew health during extended missions beyond Earth^17–19,33,39^.

### Lipidomic analysis of *Chlorella vulgaris* under simulated Earth, Moon, and microgravity conditions

**Fig. 8:**
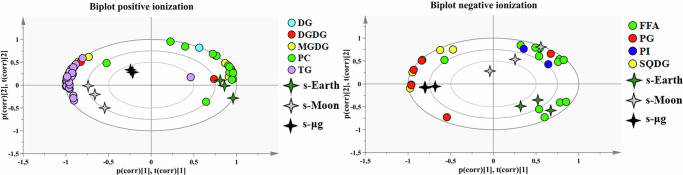
Principal Component Analysis (PCA) biplot of the lipidomic profiles of *Chlorella vulgaris* cultivated under simulated Earth (s-Earth), Moon (s-Moon), and microgravity (s-μg) conditions. For positive ionization mode two principal components explained 94% (R^2^X 0.94 and Q^2^X = 0.86) of the total variance in our dataset (PC1 = 80%, R^2^X = 0.80 and Q^2^X = 0.73, PC2 = 14%, R^2^X = 0.14 and Q^2^X = 0.49). For negative ionization mode, two pcs together explained 85% (R^2^X 0.85 and Q^2^X = 0.65) of the total variance (PC1 = 53%, R^2^X = 0.53 and Q^2^X = 0.29, PC2 = 31%, R^2^X = 0.31 and Q^2^X = 0.51).

To explore the impact of simulated gravity conditions on the lipidome of *C. vulgaris*, we performed an untargeted LC-QTOF/MS analysis under both positive and negative ionization modes (Supplementary Table 1). A total of 41 lipid species were analyzed and distributed among eight classes: phosphatidylinositols (PI), sulfoquinovosyldiacylglycerols (SQDG), phosphatidylglycerols (PG), monogalactosyldiacylglycerols (MGDG), digalactosyldiacylglycerols (DGDG), phosphatidylcholines (PC), diacylglycerols (DG), and triacylglycerols (TG). This distribution is consistent with our previous work on the lipid composition of *C. vulgaris* exposed to space radiation, where a similar set of structural and storage lipids was annotated under stress conditions^69^. A Principal Component Analysis (PCA) was carried out to assess the global lipidomic variations across gravity conditions. As shown in Fig. 8, representing positive and negative ionization modes, respectively, PCA score plots revealed a clear separation among samples grown under simulated s-Earth, s-Moon, and s-μg conditions.

Specifically, s-Earth samples clustered distinctly from both s-Moon and s-μg samples along PC1 and PC2 axes, while s-Moon and s-μg formed separate groups, suggesting that gravity reduction exerts a strong and specific effect on the lipidomic profile of *C. vulgaris*. This segregation indicate targeted physiological adjustments in lipid metabolism under altered gravity. Loading plots (Fig. 8) indicate that the most influential lipids driving this separation are TG, monogalactosyldiacylglycerols (MGDG), and phosphatidylcholines (PC), along with specific contributions from SQDG and PG. In contrast, DG and phosphatidylinositols (PI) were more associated with s-Earth samples, contributing to their distinct clustering.

These results are consistent with a profound remodeling of the lipid profile of *C*. *vulgaris*, reflecting a complex physiological adaptation to different gravity levels (Supplementary Fig. 2).

**Fig. 9:**
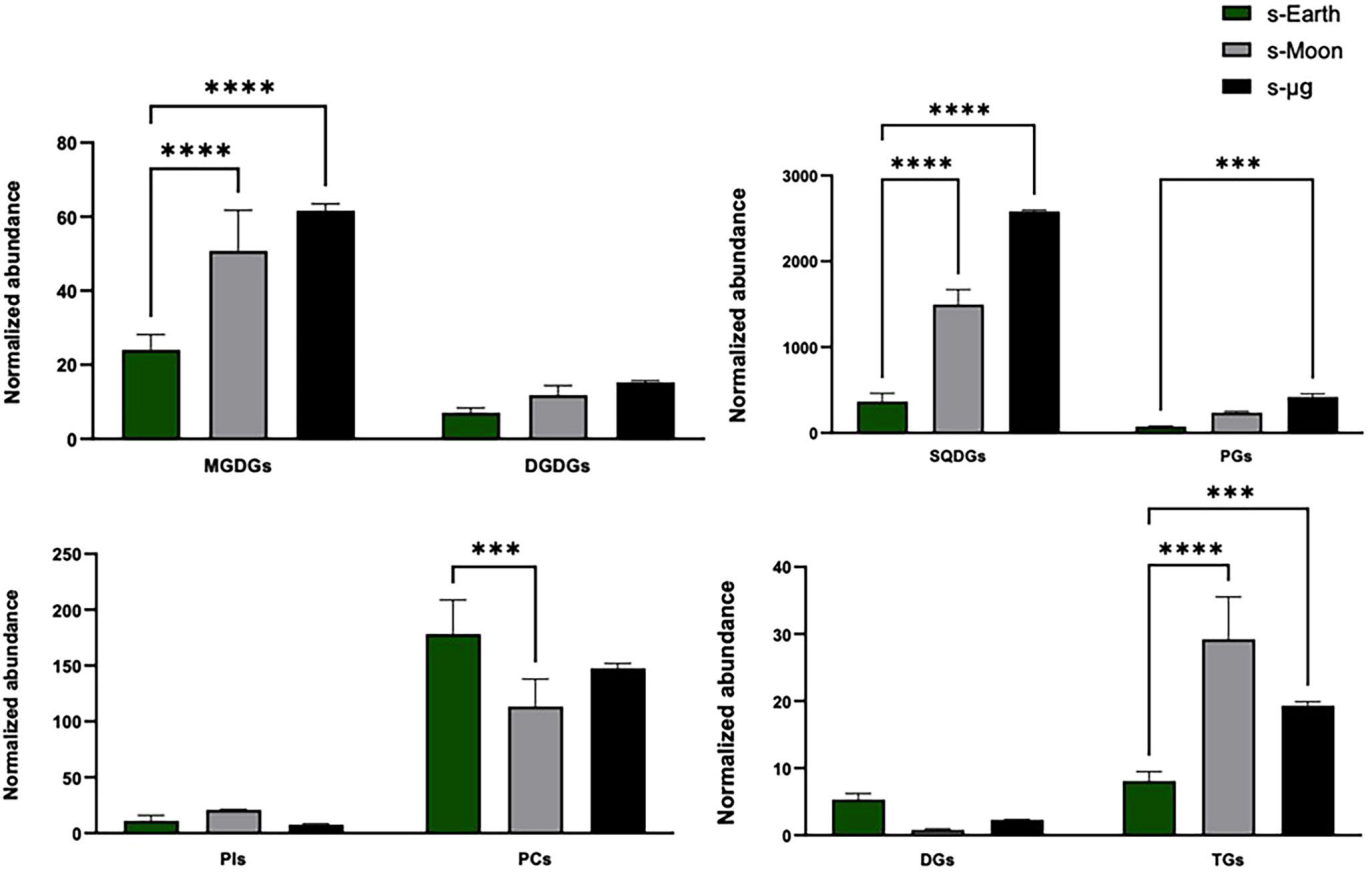
Characterization and comparison of key lipid classes obtained from the separation of *Chlorella vulgaris* samples cultivated under simulated Earth (s-Earth), Moon (s-Moon), and microgravity (s-μg) conditions. Lipid classes are as follows: diacylglycerols (DG), digalactosyldiacylglycerols (DGDG), monogalactosyldiacylglycerols (MGDG), phosphatidylcholines (PC), triacylglycerols (TG), phosphatidylglycerols (PG), phosphatidylinositols (PI), and sulfoquinovosyldiacylglycerols (SQDG). Lipid functional classes are as follows: structural lipids as MGDG, SQDG, PG, and PC; intermediates of complex lipid synthesis as DG; cell signaling lipids as PI. Bars indicate mean ± SD (*n* = 3). Significant differences across gravity conditions are denoted by asterisks (*p < 0.05, **p < 0.01, ***p < 0.001, ** ** p < 0.0001).

The analysis revealed a significant increase in structural lipids, particularly MGDG, SQDG, and PG under reduced gravity conditions (s-Moon and s-μg) compared to Earth gravity (s-Earth) (Fig. 9). These lipids, essential components of cellular and thylakoid membranes, are fundamental for maintaining membrane fluidity, integrity, and function, especially under environmental stress^78^. Among these, MGDG and SQDG were particularly increased under space-like conditions, with some species (e.g., MGDG 34:2, 34:3, 36:5 and SQDG 34:1, 34:3) showing 4- to 6-fold increases compared to Earth gravity levels. This lipid remodeling suggests a targeted adaptation aimed at preserving thylakoid membrane organization and maintaining proper photosystem function in altered gravity, in line with the known role of these glycolipids in stabilizing photosynthetic complexes^69,77,83,84^. Similarly, PG, a key phospholipid of thylakoid membranes, showed significant increases, especially PG 34:1 and PG 34:2, confirming its role in stabilizing photosystems and maintaining ionic balance. Moreover, the increase in PC, especially long and unsaturated species (e.g., PC 36:2, 36:4, 36:5), suggests that membrane remodeling in response to reduced gravity affects not only photosynthetic compartments but also cellular membranes, ensuring structural integrity and cellular functionality^85^. Importantly, several of these membrane lipids, such as PC and MGDG, are known to contain unsaturated fatty acid chains with potential antioxidants and anti-inflammatory properties, contributing to the nutritional and health-promoting value of *C. vulgaris* biomass for human consumption^86^. Alongside the increase in structural lipids, a significant accumulation of TG was observed in s-Moon and s-μg conditions, with the highest peak recorded under lunar gravity. TGs represent long-term energy reserves, and their accumulation is a typical response of microalgae to environmental stress, functioning both as energy storage and as a mechanism to sequester excess reducing power derived from photosynthetic activity^39,71^. This observation suggests an adaptive strategy aimed at energy storage, potentially essential for sustaining metabolism under reduced gravity. Moreover, TG accumulation may also play a photoprotective role, acting as an electron sink to dissipate excess absorbed energy and thus prevent the formation of harmful ROS^43,71^. From a nutritional perspective, TGs enriched in mono- and polyunsaturated fatty acids, such as oleic (C18:1) and linoleic acid (C18:2), may serve as valuable dietary lipids for astronauts, supporting cardiovascular health, inflammation control, and energy balance during long-term space missions^79,87^.

Interestingly, this response appears to align with previous findings in other microalgae, such as *Anabaena sp. PCC7120* and *Synechococcus 7942*, which under microgravity simulated on a 2D clinostat rotating at low speeds up to 7 days showed increased lipid peroxidation and enhanced membrane permeability, likely due to oxidative stress-induced membrane destabilization^44,45^. This supports the hypothesis that microgravity triggers membrane stress and lipid remodeling as a conserved adaptive mechanism among different microalgae species. In *C. vulgaris*, however, the observed increase in specific structural lipids (e.g., SQDG, MGDG, PG) may represent a protective countermeasure aimed at stabilizing membranes and mitigating peroxidative damage, thus contrasting the detrimental effects observed in other cyanobacteria under similar conditions^83,84,88,89^. In this sense, the lipid profile induced by reduced gravity not only reflects an adaptation but may also enhance the functional and nutraceutical potential of *C. vulgaris* as a dietary supplement.

However, despite the increase in TG, total lipid content in biomass was found to be reduced under s-Moon and s-μg conditions compared to Earth gravity. This apparent discrepancy can be explained by considering that *C. vulgaris*, in response to reduced gravity, selectively remodels its lipid composition: on one hand, enhancing the synthesis of specific functional lipids (such as TG and structural glycolipids essential for membranes), while on the other hand reducing other non-essential lipid fractions or membrane lipids associated with rapid cellular proliferation^71,77,85,89^. This reduction may reflect a lower requirement for new membrane synthesis under more controlled or altered growth conditions, alongside increased production of specialized lipids. Furthermore, the significant increase in carbohydrates observed under the same conditions suggests a metabolic strategy aimed at favoring more readily mobilizable and immediately available energy reserves compared to lipids, likely to meet specific bioenergetic and survival needs imposed by reduced gravity. In this context, TG accumulation emerges as a specific and targeted response, functioning as a temporary sink for reducing power and as a protective mechanism against oxidative damage, while the overall lipid balance in biomass shifts in favor of more flexible carbon reserves, such as carbohydrates^43,58,75,76^.

A key element to understanding this lipid reorganization is its connection to mitochondrial metabolism, extensively documented in *C. vulgaris* exposed to simulated microgravity. Popova^43^ demonstrated that under clinorotation conditions, *C. vulgaris* cells exhibit a significant increase in mitochondrial volume, cristae organization, and matrix density, accompanied by enhanced activity of Mg^2+^-ATP synthase and SDH^43^. These changes suggest that, in response to reduced gravity, *C. vulgaris* boosts its mitochondrial respiratory pathway, likely to ensure sufficient energy production and to counteract oxidative stress associated with excess NADPH and reducing power^27,43,90,91^. In this context, the observed remodeling of membrane lipids, particularly the increase in PG, PC, and SQDG, may reflect the need to stabilize not only photosynthetic membranes but also mitochondrial membranes, ensuring structural support for intensified respiratory activity^43,83,84,92^. Likewise, TG accumulation may serve as a secondary metabolic balancing mechanism, useful to sequester excess acetyl-CoA and NADPH generated by hyperactive respiration^93–95^. Therefore, a possible synergy between lipid remodeling and mitochondrial metabolism emerges, aimed at maintaining cellular energetic and structural homeostasis under space-like conditions.

In contrast to TG, DG, key intermediates in the synthesis of complex lipids, were significantly reduced in lunar and microgravity conditions compared to Earth, indicating a possible shift in lipid metabolism toward the direct accumulation of storage lipids (TG) and maintenance of essential structural lipids, rather than ongoing membrane lipid biosynthesis^43,69,71^. This could be related to a lower rate of cell division or altered metabolic priorities under gravitational stress. Similarly, phosphatidylinositols (PI), known for their role in cell signaling, showed globally low and stable levels, although some species (PI 34:1 and PI 34:2) were selectively increased under s-Moon, suggesting a possible involvement in gravity-specific signaling mechanisms^69,78^. Although present in low amounts, some PI species may also contribute to the bioactive lipid pool, with potential signaling or immunomodulatory functions relevant for human health, a topic worth exploring in future research^96^.

The analysis of the UI across different lipid classes revealed a selective and condition-specific remodeling of fatty acid unsaturation in *Chlorella vulgaris* under reduced gravity. Although no statistically significant changes were detected, MGDG shows a gradual increase in UI from s-Earth (58 ± 10) to s-Moon (75 ± 16) and s-μg (100 ± 3), indicating a tendency toward higher acyl-chain desaturation under reduced gravity. This trend, together with the rise in normalized abundance, suggests a mild activation of galactolipid desaturation, possibly mediated by plastidial desaturases, as previously reported under stress conditions^97,98^. The simultaneous increase in abundance and UI supports the role of MGDG in tuning thylakoid membrane fluidity and curvature to preserve photosynthetic efficiency in altered gravitational environments. While no significant changes were observed in MGDG and DGDG UIs despite their increased abundance, a relevant rise in UI was detected in SQDG and PG, particularly under s-μg compared to s-Earth. Specifically, UI values for SQDG increased more than 20-fold (from 73 ± 36 to 1533 ± 19), and for PG by over 9-fold (from 40 ± 16 to 374 ± 28), indicating a strong enrichment in polyunsaturated acyl chains (Supplementary Table 3). These findings are consistent with previous reports demonstrating the critical role of SQDG and PG in thylakoid membrane function and stability, particularly under environmental stress conditions^83,84^. The increase in polyunsaturated forms of these lipids may serve to counterbalance the rigidity induced by microgravity, ensuring the fluidity and curvature required for optimal photosystem organization and function^83^. In particular, PG is essential for the stabilization of the oxygen-evolving complex in Photosystem II, and its remodeling likely reflects a protective strategy to maintain photosynthetic efficiency in altered gravitational conditions^84^.

Interestingly, PC showed a significant decrease in both normalized abundance (Fig. 9) and unsaturation index (UI) (Supplementary Table 3) under s-Moon, with partial recovery at s-μg, suggesting that extraplastidic membranes respond differently to intermediate gravitational cues, potentially through stress-induced modulation of desaturase activity or altered membrane turnover rates. These differences in unsaturation trends among lipid classes potentially support that lipid remodeling is a compartmentalized and lipid-specific response^77^. Lipid classes, such as PI, DG, and TG maintained relatively stable UI values, indicating that their function, linked to signaling, intermediate metabolism, and energy storage, is less dependent on desaturation under gravity variation compared to PC. The consistent UI of DG and the concurrent increase in TG species enriched in unsaturated fatty acids suggest a redirection of intermediates toward energy storage rather than ongoing membrane biogenesis, supporting TG role as a photoprotective and redox-buffering reservoir^43,71^. Overall, these results suggest that *C. vulgaris* could remodel lipid desaturation pathways to preserve critical membrane functions under reduced gravity. This adaptation, focused on chloroplast and mitochondrial membranes, likely supports both photosynthetic performance and respiratory activity, aligning with mitochondrial structural adaptations previously reported under clinorotation^43^.

Taken together, lipidomic data indicates that *C. vulgaris* responds to reduced gravity through a profound reorganization of both its lipid and mitochondrial metabolism: on the one hand, enhancing the synthesis of structural lipids to preserve membrane integrity and functionality; on the other hand, accumulating TG as an energy reserve and cellular protection strategy. These adaptations are consistent with stress responses observed in other microalgae and reflect considerable metabolic plasticity of *C. vulgaris*. Furthermore, these data complement the broader metabolic picture observed in this study, including increased biomass productivity, enhanced photosynthetic pigment content, and higher antioxidant capacity under reduced gravity. Collectively, these findings underscore the suitability of *C. vulgaris* for BLSS in space missions^59,70^. Finally, the accumulation of TG and specific unsaturated membrane lipids may enhance the nutritional value of *C. vulgaris* biomass, representing a source of beneficial fatty acids for astronaut nutrition, while also supporting in situ biofuel production strategies, contributing to the sustainability of future space habitats.

### Practical implications for astronaut nutrition and bioreactor design

Using the highest biomass productivity obtained, we evaluated the feasibility of meeting astronauts’ daily nutritional needs with *C. vulgaris* biomass cultivated under space-like conditions. Microalgae could be grown in various photobioreactor configurations, including open pond systems within pressurized domes, as described by Cao et al.^22^. We estimated the daily biomass required to supply proteins, carbohydrates, and lipids for a six-member crew, along with the corresponding minimum culture volumes under different gravity simulations (Table 2).

**Table 2:** Estimated daily biomass requirement and minimal culture volume to meet protein, carbohydrate, and lipid needs for a crew of six astronauts, based on *Chlorella vulgaris* cultivated under simulated Earth (s-Earth), Moon (s-Moon), and microgravity (s-μg) conditions

Nutrient	Condition	Nutrient’s need (g kg^−1^ day^−1^)	Daily need per astronaut (g day^−1^)	Daily need for 6 astronauts (g day^−1^)	*Chlorella* content (g g^−1^)	Biomass needed (g day^−1^)	ΔX (g L^−1^ day^−1^)	Volume of culture (m^3^)
	s-Earth	1.04	78	468	0.40	1.169 × 10^3^	0.22	5.31
Proteins	s-Moon				0.28	1.656 × 10^3^	0.26	6.37
	s-μg				0.31	1.498 × 10^3^	0.26	5.77
	s-Earth	0.78	55.5	351	0.18	1.984 × 10^3^	0.22	9.02
Carbohydrates	s-Moon				0.34	1.035 × 10^3^	0.26	3.98
	s-μg				0.34	1.043 × 10^3^	0.26	4.01
	s-Earth	0.86	64.5	387	0.14	2.843 × 10^3^	0.22	12.93
Lipids	s-Moon				0.11	3.654 × 10^3^	0.26	14.05
	s-μg				0.09	4.225 × 10^3^	0.26	16.25

Nutritional needs from Bychkov et al.^120^.

As shown in Table 2, to meet the daily protein requirements for six astronauts, the biomass needed ranges from 1.169 × 10^3 ^g day^−1^ (s-Earth) to 1.656 × 10^3 ^g day^−1^ (s-Moon), corresponding to photobioreactor volumes between 5.31 and 6.37 m^3^. The increased carbohydrate content observed under s-Moon and s-μg conditions allows a reduction in the biomass required to meet daily carbohydrate intake, down to approximately 4 m^3^ under reduced gravity. Conversely, fulfilling lipid needs would require substantially larger culture volumes, up to 1.625 × 10^4 ^L day^−1^ (16.25 m^3^) under s-μg, due to the reduced lipid content observed in microgravity.

Although the culture volumes required for *C. vulgaris* biomass production are technically achievable within space habitats, the maximum safe intake of this biomass must be carefully controlled, primarily due to the risk of excessive carotenoid-derived vitamin A intake. According to NASA guidelines, vitamin A consumption should not exceed 3000 μg per day to prevent toxicity^99^. Based on measured carotenoid concentrations, the safe daily intake of *C. vulgaris* would be approximately 14.25 g day^−1^ under s-Earth, but decreases significantly under reduced gravity, reaching 5.05 g day^−1^ in s-Moon and 4.38 g day^−1^ in s-μg, due to carotenoid accumulation in these conditions.

Nonetheless, considering these limits and drawing from recent analyses of commercial microalgal supplements, which indicate that daily doses up to 10 g day^−1^ are commonly used and considered safe (link), a pragmatic daily intake of 10 g of *C. vulgaris* biomass is proposed. This dose would provide approximately 1.5 g of polyphenols (nearly 100% of daily antioxidant needs), around 3.1 g of protein (covering ~4% of daily protein requirements), and a controlled carotenoid intake^33,36,59^. Although insufficient as a primary macronutrient source, this amount allows *C. vulgaris* to function as a nutraceutical and antioxidant supplement, critical for mitigating oxidative stress during long-duration space missions.

From an operational standpoint, maintaining this intake would require about 38-40 L day^−1^ of culture volume, assuming an average productivity of 0.26 g L^−1^ day^−1^, a manageable demand when distributed across 4–5 modular photobioreactors, ensuring redundancy and resilience of the system.

Thus, a daily intake of 10 g of *C. vulgaris* emerges as a feasible and effective functional food supplement for astronauts, capable of enhancing antioxidant defenses, supporting psychological well-being, and contributing to oxygen production within BLSS. Although *Chlorella* alone cannot meet daily macronutrient needs, its role as a functional supplement, covering 3–4% of protein requirements, would significantly aid in maintaining crew health. If the goal were to meet 30–40% of protein demands, larger culture volumes (2–2.5 m^3^ under s-Moon and s-μg conditions) would be necessary. Future experiments in real spaceflight environments will be essential to validate these projections and to optimize bioreactor design, especially regarding gas exchange, mixing, and light management in microgravity.

While this study may indicate physiological and biochemical responses of *Chlorella vulgaris* under simulated lunar and microgravity conditions, several methodological constraints inherent to ground-based simulation should be acknowledged. The RPM randomizes the gravity vector, functionally reproducing microgravity without replicating the exact physical conditions of spaceflight. Similarly, the simulated lunar gravity (0.17 g) represents a kinematic approximation rather than a true partial-gravity environment. Cultures were grown in completely filled, gas-permeable T-25 flasks, a configuration that prevents bubble formation and shear artifacts but limits active gas exchange, potentially generating localized CO_2_ and O_2_ gradients. Photosynthetic performance was inferred indirectly from pH and growth dynamics, as no direct fluorescence or oxygen evolution measurements were performed. Biological replicates were cultivated simultaneously on the same device, ensuring identical mechanical conditions but preventing complete spatial randomization across treatments. The experiments were also conducted on a small scale and over a limited period (7 days), restricting extrapolation to long-term bioregenerative systems. Despite these limitations, the setup ensured comparable mechanical conditions across gravity treatments and yielded physiological responses consistent with those reported in previous studies, supporting the biological relevance of the model and its value for future space bioprocess applications^6,9,40,43,44,63,100–102^.

**Fig. 10:**
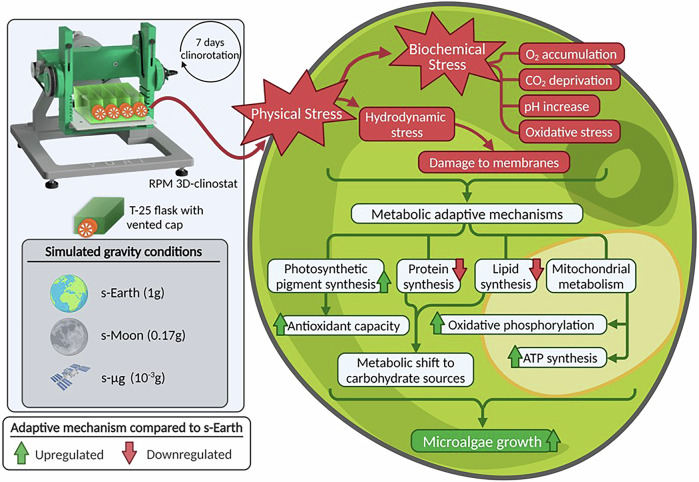
Summary of the effects and adaptive mechanisms of *Chlorella vulgaris* in simulated gravity conditions. Physical and biochemical stress effects induced by simulated gravity (s-Moon and s-μg) conditions are summarized as well as the metabolic mechanisms adopted by *Chlorella vulgaris* to sustain its growth. The graphical summary is based on the evidence obtained from this work and from others^40,43^. Created with BioRender.com.

Overall, this study provides novel insights into the physiological, biochemical, and lipidomic adaptations of *C. vulgaris* cultivated under simulated terrestrial, lunar, and microgravity conditions, with potential relevance to BLSS. Our findings demonstrate that reduced gravity environments significantly enhance biomass productivity, photosynthetic pigment accumulation, and antioxidant capacity (summarized in Fig. 10). Specifically, *C. vulgaris* cultivated under lunar and microgravity conditions exhibited substantial increases in chlorophylls, carotenoids, and polyphenols, indicating that gravitational stress triggers a distinct metabolic reprogramming aimed at improving resilience and biosynthetic efficiency.

These adaptive responses suggest that *Chlorella vulgaris* could maintain metabolic activity and nutritional value under altered gravity, supporting its potential relevance as a microalgal species for next-generation BLSS. Future research should prioritize the optimization of large-scale cultivation under real and partial gravity conditions, the refinement of photobioreactor design, and the assessment of long-term physiological stability to facilitate its integration into circular bioeconomy frameworks for future space exploration and habitation.

## Methods

### Strain maintenance and pre-cultivation conditions

*Chlorella vulgaris* CCALA 269 was obtained from the Culture Collection of Autotrophic Organisms (CCALA) in Třeboň, Czech Republic. The strain was routinely maintained in Bold Basal Medium (BBM) at 25 °C within a 5-L glass flask. Illumination was provided at an intensity of 100 μmol photons m^−2^ s^−1^ (Delta Ohm HD2102.1 portable luxmeter, GHM GROUP, Germany) under a 12 h light / 12 h dark cycle. The cultures were continuously shaken using a Stuart SSM1 Orbital Shaker (Biosigma, Italy) set to 100 rpm.

### Experimental setup for cultures under simulated reduced gravity

To investigate whether the growth and nutritional profile of *C. vulgaris* could be influenced by different levels of simulated gravity (Earth, Moon, and microgravity), a 3D clinostat (Random Positioning Machine, RPM 2.0, Yuri Gravity) was employed (Fig. 1). This device enables the simulation of altered gravity conditions, including terrestrial gravity (1 g), lunar gravity ( ~ 0.17 g), and microgravity ( ~ 0.001 g). The clinostat operates based on the principle of gravitational vector averaging, utilizing two independently rotating perpendicular frames to continuously modify the orientation of the gravitational vector relative to the sample. This dynamic reorientation effectively minimizes the unidirectional influence of gravity, creating a condition analogous to microgravity^50,101,103^. To avoid biases from sedimentation, light-shading, and variable shear forces, we employed the clinostat at 1 g as our Earth gravity control. This approach maintains identical mixing dynamics and light exposure across all treatments, isolating the effect of reduced gravitational acceleration (0.17 g and 0.001 g) on *Chlorella vulgaris* physiology.

The experiments were conducted in batch culture within transparent, vented T-25 flasks (Corning^®^, Merk Life Science Milano, Italy), each filled with ~80 mL of Bold’s Basal Medium (BBM). To prevent shear stress artifacts, any air bubbles were carefully removed prior to incubation^50,51^. Each T-25 flask was equipped with a gas-permeable filter cap that enabled passive O_2_/CO_2_ diffusion during clinorotation while preventing contamination and fluid leakage. The cultures were illuminated with white light at an intensity of 100 μmol photons m^−2^ s^−1^, measured at the flask surface, under a 12 h light/dark photoperiod. The entire system was maintained in a temperature-controlled incubator set at 30 °C to ensure optimal growth conditions.

At the beginning of each experiment, the OD (OD_650_) was standardized to ~0.2, ensuring uniform starting conditions across replicates. Each experimental condition (1 g, 0.17 g, and 0.001 g) was assessed in quadruplicate.

Each simulated gravity condition was performed with four independent biological replicates inoculated from the same exponential-phase mother culture of *Chlorella vulgaris*, standardized for OD and pH, using identical rotation profiles and environmental parameters across all runs. Preliminary validation runs confirmed reproducible growth kinetics and pH evolution under identical RPM settings, ensuring biological consistency among experimental conditions.

The clinostat was interfaced with a computer-controlled software system, allowing precise modulation of rotation speed and motion patterns to maintain the desired gravity simulation. The microalgae were subjected to the simulated altered gravity conditions for a total duration of 7 days.

To monitor growth dynamics and metabolic activity, OD (OD_650_) and pH measurements were recorded daily using a Hanna Instruments HI 2210 pH meter (Hanna Instruments, Woonsocket, RI, USA). Daily samples (0.5 mL) were aseptically withdrawn and reintroduced through the vented cap using sterile syringes to maintain constant volume and avoid air bubble formation.

### Determination of biomass and growth parameters

To establish a calibration curve for biomass estimation (Supplementary Fig. 1), seven sampling points were selected, covering an OD range from 1.2 to 0.2. Each measurement was performed in quadruplicate. For each point, 10 mL of diluted culture was collected and centrifuged at 4000 rpm for 15 min to separate the biomass from the medium. The resulting pellet was rinsed with Milli-Q water to remove any residual culture medium before being transferred to a pre-weighed tube. To determine the dry biomass concentration, the pellets were dried in an oven at 60 °C for 48 ours, or until a constant weight was reached. The total weight of the tube, including the dried biomass, was recorded as *W*_*2*_, while the initial weight of the empty tube was noted as *W*_*1*_. The difference between these two values (*W*_*2*_ - *W*_*1*_) provided the dry biomass mass, which was then normalized to 10 mL of culture to obtain the biomass concentration in mg mL^−1^. These values were subsequently plotted against OD (650 nm) to generate a regression equation, which allowed for the conversion of OD measurements into biomass concentrations throughout the experiment. Due to practical constraints on culture volume, a single OD_650_ calibration curve was applied across all simulated gravity conditions.

The dry biomass concentration (*X*), expressed in g L^−1^, was calculated using the following Eq. 1:

1 $$X=\frac{{W}_{2}-{W}_{1}}{V}$$

Where, *V* represents the volume of the sampled culture.

The average biomass productivity (Δ*X*) was determined as the ratio between the maximum biomass concentration (*X*_*max*_) and the time required to reach this value (*t*_*max*_). This parameter provides insights into the overall efficiency of biomass production over time and was calculated using the following Eq. 2:

2 $$\Delta X=\frac{{X}_{\max }}{{t}_{\max }}$$

Where, *X*_max_ is the maximum biomass concentration (in g L^−1^) measured at time *t*_max._

To further assess the microbial growth dynamics, the specific growth rate (*μ*) was calculated, representing the rate at which the biomass increases per unit of time during the exponential growth phase. This was determined using the following Eq. 3:

3 $$\mu =\frac{{\mathrm{ln}}\left({X}_{2}\right)-{\mathrm{ln}}\left({X}_{1}\right)}{{t}_{2}-{t}_{1}}$$

Where, *X*_*2*_ and *X*_*1*_ correspond to the dry biomass concentrations (in g L^−1^) at times *t*_*2*_ and *t*_*1*_, respectively.

### Chemicals

Eur.-Reag grade reagents, specifically sulfuric acid (96%), orthophosphoric acid (85%), sodium nitrate, potassium chloride, phenol, copper sulfate, sodium hydroxide, and sodium potassium tartrate, were sourced from Sigma-Aldrich (Merck KGaA, Darmstadt, Germany). Additional RPE-ACS-for analysis-reag. Ph. eur.-reag chemicals were obtained from Carlo Erba (Valde Reuil Cedex, France). Meanwhile, sodium carbonate and the Folin–Ciocalteau reagent were procured from Sigma-Aldrich Inc. (St. Louis, MO, USA), and glucose, bovine serum albumin, as well as vanillin standards were purchased from Sigma-Aldrich (Merck KGaA, Darmstadt, Germany). Ultrapure water with a conductivity below 18.2 MΩ was generated using a Milli-Q system (Millipore, Milan, Italy).

The SPLASH^®^ LIPIDOMIX^®^ standard mixture, containing a defined set of lipid components, was obtained from Sigma Aldrich (Milan, Italy). This mix includes PC (15:0–18:1) (d7), PE (15:0–18:1) (d7), PS (15:0–18:1) (d7), PG (15:0–18:1) (d7), PI (15:0–18:1) (d7), PA (15:0–18:1) (d7), LPC (18:1) (d7), LPC 25, LPE (18:1) (d7), Cholesteryl Ester (18:1) (d7), MG (18:1) (d7), DAG (15:0–18:1) (d7), TG ((15:0–18:1) (d7)-15:0), SM (18:1) (d9), and deuterated Cholesterol (d7).

### Sample Preparation for Chemical Characterization Analysis

Cultures were harvested at the end of the experiment (7th day) by centrifugation at 4000 rpm for 10 min at 20 °C. The supernatant was carefully discarded, and the pellet was washed three times with Milli-Q water to remove any residual medium. The washed cell pellet was subsequently frozen at -20 °C, lyophilized using a Lyovapor L-200 freeze-dryer (Buchi, Milan, Italy), and ground into a fine powder using a mortar and pestle. The dried biomass was stored in the dark inside a vacuum desiccator until further analysis. Three independent replicates were performed for each experimental condition.

### Determination of total carbohydrates, lipids, and total soluble proteins

Macronutrients were quantified following the methodology used by Fais et al.^68^. Carbohydrate (C) content was determined using the modified Dubois et al.^104^ protocol, while total lipids (TL) were measured according to Chen and Vaidyanathan^105^ and Bligh and Dyer^106^. Protein concentration (TSP) was assessed following Lowry et al.^107^. All samples were analyzed with a Multiskan SkyHigh microplate spectrophotometer (Thermo Fisher Scientific), employing external standards for quantification. The results, expressed in mg kg^−1^ are reported as the mean ± standard deviation (SD) of triplicate measurements.

### Determination of chlorophyll and total carotenoids

Approximately 2 mg of *C. vulgaris* biomass dry powder was suspended in 1.5 mL of 96% ethanol and subjected to ultrasonication for 1 min at 80% power using a Bandelin Sonoplus HD 4100 ultrasonic rod (Bandelin Electronic GmbH & Co. KG, Germany), then vortexed and incubated in a boiling water bath for 15 min. The suspension was then centrifuged at 4000 rpm for 5 min. Absorbance of supernatants were recorded at 720 nm (baseline correction), 665 nm (chlorophyll a), 649 nm (chlorophyll b), and 470 nm (total carotenoids), using 70% methanol as the blank. Pigment concentrations were determined according to the correlations proposed by Ritchie^108^ for total carotenoids and Wellburn^109^ for chlorophylls. All spectrophotometric analyses were performed using a Multiskan SkyHigh microplate spectrophotometer (Thermo Fisher Scientific). The results were expressed as the mean ± SD of triplicate measurements.

### Determination of total polyphenols

The total polyphenol content was measured using the Folin-Ciocalteu assay, according to a modified protocol based on Singleton and Rossi^110^. Briefly, 100 μL of each sample extract or gallic acid standard were mixed with 500 μL of Folin-Ciocalteu reagent and incubated for 5 min at room temperature. Subsequently, 3 mL of 10% (w/v) Na₂CO₃ solution and ultrapure water were added to reach a final volume of 10 mL. Following a 90 min incubation at room temperature, absorbance was recorded at 725 nm against a blank using a Multiskan SkyHigh microplate spectrophotometer (Thermo Fisher Scientific). Quantification was carried out using an external calibration curve (gallic acid standard), and results were expressed as mg kg^−1^ gallic acid equivalents (GAE).

### Determination of Antioxidant Power

The method reported by Brand-Williams et al.^111^ was adopted for this study. Approximately 2 mg of lyophilized *C. vulgaris* powder was dispersed in 500 μL of methanol and sonicated for 1 min at 80% power using a Bandelin Sonoplus HD 4100 ultrasonic rod (Bandelin Electronic GmbH & Co. KG, Germany). The mixture was then centrifuged at 4000 rpm for 5 min, and 50 μL of the resulting supernatant was combined with 2 mL of a methanolic DPPH solution (50 μmol) to determine total (or standard) polyphenol levels. After 60 min of incubation, absorbance was measured at 517 nm using a Multiskan SkyHigh microplate spectrophotometer (Thermo Fisher Scientific). Quantitative analysis was performed via the external standard method (Trolox), relating the absorbance value to concentration. Results are expressed in mmol kg^−1^ TEAC (Trolox equivalent antioxidant capacity).

### Sample preparation for lipidomics analysis and complex lipid analysis

Lyophilised *C. vulgaris* samples were extracted using a slight modification of the method by Folch et al.^112^. Briefly, 10 mg of each lyophilised biomass sample was transferred to a centrifuge tube and 10 mL of bi-distilled water was added. Solutions were then ultra-sonicated for 3 min using ExtractorOne (GM solution, Cagliari, Italy). Subsequently 5000 μL of methanol/chloroform (1/2 v/v) and 10 μL of the internal standards (Succinic acid-2,2,3,3-d4) were added. Solutions were agitated every 15 min for 1 h. After 1 h, 1000 μL of aqueous 0.2 M potassium chloride were added and the samples were agitated again. Each sample was centrifuged at 24104 rcf for 10 min. After centrifugation, 1000 μL of lipophilic layers were transferred into distinct glass vial and dried with a gentle nitrogen stream.

The dried lipophilic phase was dissolved in a mixture of methanol/chloroform (1:1 v/v, 20 μL) and diluted with a mixture of 2-propanol/acetonitrile/water (2:1:1 v/v/v, 380 µL) containing the internal standard Cer(d18:1/25:0). Complex lipids were analyzed using a UHPLC-QTOF/MS coupled with an Agilent 1290 Infinity II LC system, with injections of 5 μL and 8 μL in the positive and negative ionization modes, respectively. Chromatographic separation was achieved using a Kinetex 5 µm EVO C18 100 A, 150 mm×2.1 μm column (Agilent Technologies, Palo Alto, CA), maintained at 50 °C and with a flow rate of 0.2 mL min^−1^. For the positive ionization mode, the mobile phase comprised (A) a 10 mM ammonium formate solution in 60% milli-Q water and 40% acetonitrile, and (B) a 10 mM ammonium formate solution in isopropanol and acetonitrile mixture (9:1 v/v). The gradient in positive ionization mode consisted of an initial 60% A, followed by a linear decrease to 50% A over 2 min, then to 1% over 5 min, maintaining this percentage for 1.9 min before returning to the initial conditions in 1 min. The mobile phase in negative ionization mode differed only in the use of 10 mM ammonium acetate instead of ammonium formate. The MS source was operated with the following parameters: gas temperature, 200 °C; gas flow (nitrogen), 10 L min^−1^; nebulizer gas (nitrogen), 50 psig; sheath gas temperature, 300 °C; sheath gas flow, 12 L min^−1^; capillary voltage, 3500 V for positive and 3000 V for negative; nozzle voltage, 0 V; fragmentor, 150 V; skimmer, 65 V; octapole RF, 7550 V; mass range, 50–1700 m/z; collision energy, 20 eV in positive and 25 eV in negative mode; mass precursor per cycle = 3; threshold for MS/MS, 5000 counts. Chromatographic areas were obtained by acquiring samples in ESI full scan mode and were normalized using PE 15:0-18:1(d7) as an internal standard. Consequently, results are expressed as a ratio to an internal standard and are referred to as normalized abundance.

### Identification and quantification of complex lipid classes

To identify lipids classes, iterative MS/MS experiments were conducted, at two different collision energies (CEs, 20 and 40 eV) to improve mass fragmentation. This method consists in injecting the same sample multiple times, while precursors previously selected for MS/MS fragmentation are excluded on a rolling basis. Four different iterative analyses were performed for maximizing the maximum number of lipid species with a mass error tolerance of 20 ppm and a retention exclusion tolerance of 0.2 min. Lipids were identified in the MS/MS spectra using the putative mass annotation provided by Lipid annotator (version 1.0, Agilent MassHunter workstation), comparing with an online mass database^113^ and analyzing the diagnostic fragment for each lipid class^114–117^. Monogalactosyldiacylglycerol (MGDG) and sulfoquinovosyldiacylglycerol (DGDG) were identified as [M + NH_4_]^+^ by using the product ion resulting from the combined neutral loss of NH_3_ and galactosyl unit (-197 Da) and for DGDG by using the neutral loss of NH_3_ and digalactosyl unit (-359 Da). Others product ions for MGDG and DGDG resulted from the loss of fatty acyl groups as acylium ions plus 74 Da [RCO + 74]^+^. Phosphatidylcholine (PC) was detected in positive ionization mode and annotated as [M + H]^+^, while the fatty acyl composition was determined in negative using the [M + CHO_2_]^−^ adduct. Diacylglycerol (DG) and triacylglycerol (TG) were detected in positive ionization mode and annotated as [M + NH_4_]^+^. Fatty acyl chains were determined using the three typical DG^+^ (or the two MG^+^ in the case of DG) ions corresponding to the loss of fatty acids (FA), after which the FA composition of TG molecule was deduced using the mass difference. Sulfoquinovosyldiacylglycerol (SQDG) was detected in negative ionization mode and identified as [M-H]^−^. The diagnostic product ion at m/z 225.0 due to the loss of the polar head was used to confirm SQDG species. The fatty acyl composition was determined using the product ions resulting from the neutral loss of the fatty acyl group as free carboxylic acid (RCOOH). Phosphatidylinositol (PI) was detected as [M-H]^−^ and confirmed using the product ions at m/z 241 (derived from the cyclic anion of inositol phosphate) and at m/z 223 (loss of water from m/z 241). Phosphatidylglycerol (PG) was determined as [M-H]^−^ and using the product ions at m/z 153 (corresponding to the cyclic phosphate anion) and at m/z 391 (resulting from the loss of fatty acyl group at sn-2 with the polar headgroup glycerol).

### Unsaturation Index

To quantify changes in lipid unsaturation under altered gravity, we calculated the UI for each lipid class following the method proposed by Hong et al. ^118^, referenced in Narayanan et al. ^119^. While the original approach uses molar percentages, in our study, molar concentrations were not available. Therefore, we calculated the UI using normalized peak areas (relative to internal standards), which have been widely used in semi-quantitative lipidomics. The formula was adapted as in the following Eq. 4:

4 $$\begin{array}{l}UI=\sum \left(Amount\,of\,lipid\,molecular\,speciesx\,\frac{number\,of\,double\,bonds\,}{number\,of\,acyl\,chains}\right)\\ \,\,\,\,=\sum \left(\frac{Area\,of\,molecular\,species}{Area\,of\,Internal\,standard}x\frac{number\,of\,double\,bonds\,}{number\,of\,acyl\,chains}\right)\end{array}$$

The UI for each lipid head group class was then obtained by summing the individual UI values of all molecular species belonging to that class.

### Statistical Analysis

All experiments were performed in triplicate, and results are presented as mean ± SD. Univariate analyses were performed with the GraphPad Prism software (version 8.3.0, Dotmatics, Boston, Massachusetts). Mean differences between groups were tested for statistical significance using ANOVA tests with Dunnett’s and Tukey’s correction for multiple comparisons. The significance levels based on the *p*-values are indicated by asterisks (*). No asterisk corresponds to a *p*-value > 0.05, * to 0.005 < *p*-value < 0.05, ** to 0.0005 < *p*-value < 0.005, *** to 0.0005 < *p*-value < 0.0001 and **** to *p* < 0.0001.

Unsupervised Principal Component Analyses (PCA) were performed for dataset overview. Results are shown in two dimensions as score (related to observations) and loading (related to variables) scatter plots. This multivariate analysis was performed with the SIMCA-P+ software (version 14.1, Umetrics, Sartorius, Germany). The quality of the models and the optimum number of principal components were evaluated based on the cumulative parameters R^2^X (goodness of fit) and their analogues in cross validation Q^2^ (goodness of prediction).

## Supplementary information

Supplementary information

## Data Availability

All data generated or analysed during this study are included in this published article and its supplementary information file.
