# Supplementary material for: Superfood potential of Chlorella vulgaris: productivity and antioxidant boost under simulated moon and microgravity conditions
Source: NPJ Microgravity. 2025 Dec 12;12:8. doi: 10.1038/s41526-025-00550-4 (PMC12800078; doi:10.1038/s41526-025-00550-4)
Supplement: Supplementary file 1 — Supplementary information [file 41526_2025_550_MOESM1_ESM.pdf]

Supplementary materials

Superfood Potential of *Chlorella vulgaris*: Productivity and Antioxidant Boost under Simulated Moon and Microgravity Conditions

Giacomo Fais[1,2], Filippo Ghiani[1,2], Debora Dessì[3], Mattia Casula[3], Giovanni Perra[1,2], Eleonora Torchia[1,2], Nicola Lai[1,2], Alessandro Concas[1,2*], and Giacomo Cao[1,2,4]

[1] Interdepartmental Centre of Environmental Science and Engineering (CINSA), University of Cagliari, Via San Giorgio 12, 09124 Cagliari, Italy
[2] Department of Mechanical, Chemical and Materials Engineering, University of Cagliari, Via Marengo 2, 09123 Cagliari, Italy
[3] Department of Life and Environmental Sciences, University of Cagliari, 09042 Cagliari, Italy
[4] Center for Advanced Studies, Research and Development in Sardinia (CRS4), Loc. Piscina Manna, Building 1, 09050 Pula, Italy

[*] Corresponding authors: alessandro.concas@unica.it; giacomo.fais@unica.it

Supplementary Figures

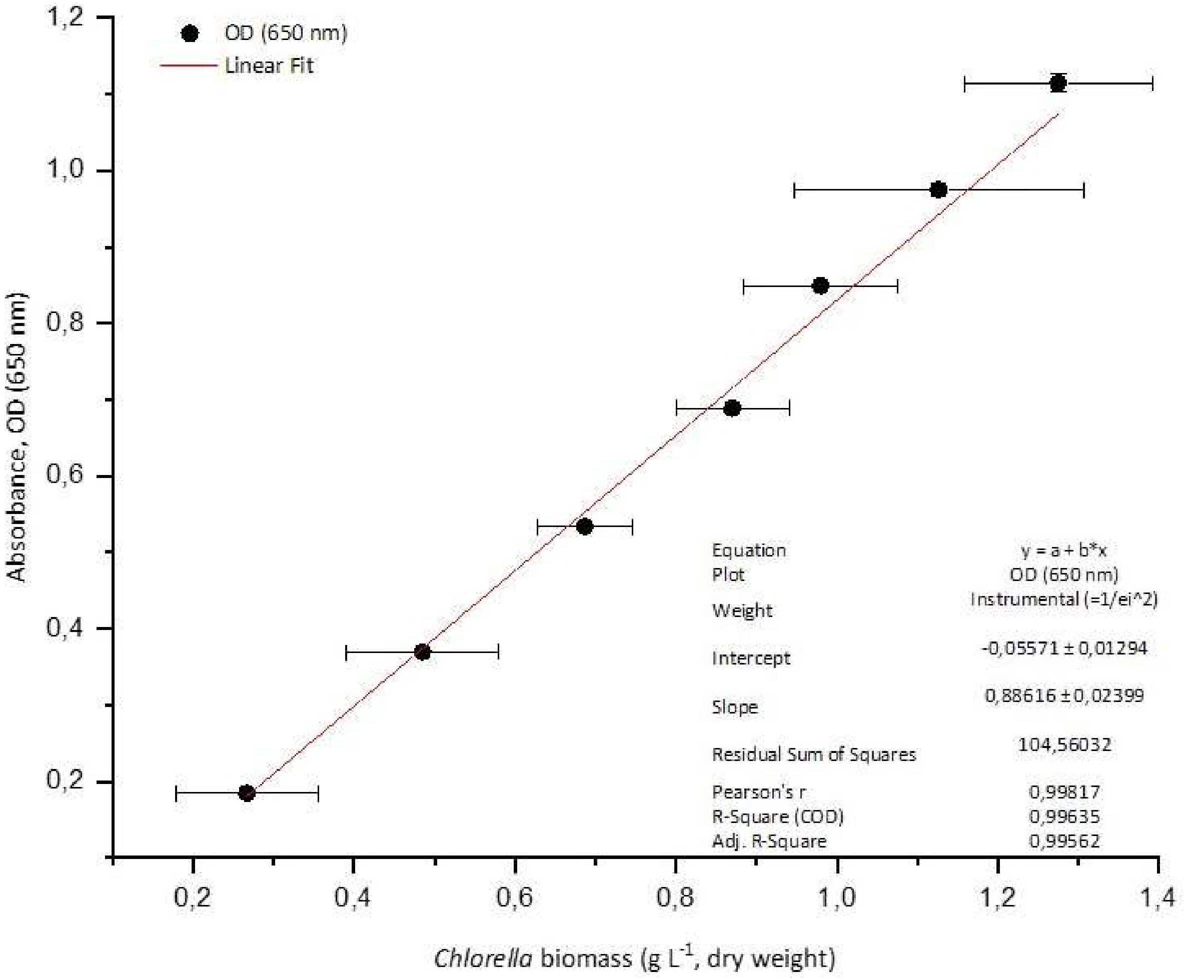

Supplementary Figure 1. Calibration curve for biomass production of *Chlorella vulgaris*. Absorbance (OD_{650}) vs. biomass concentration (g L^{-1}, dry weight).

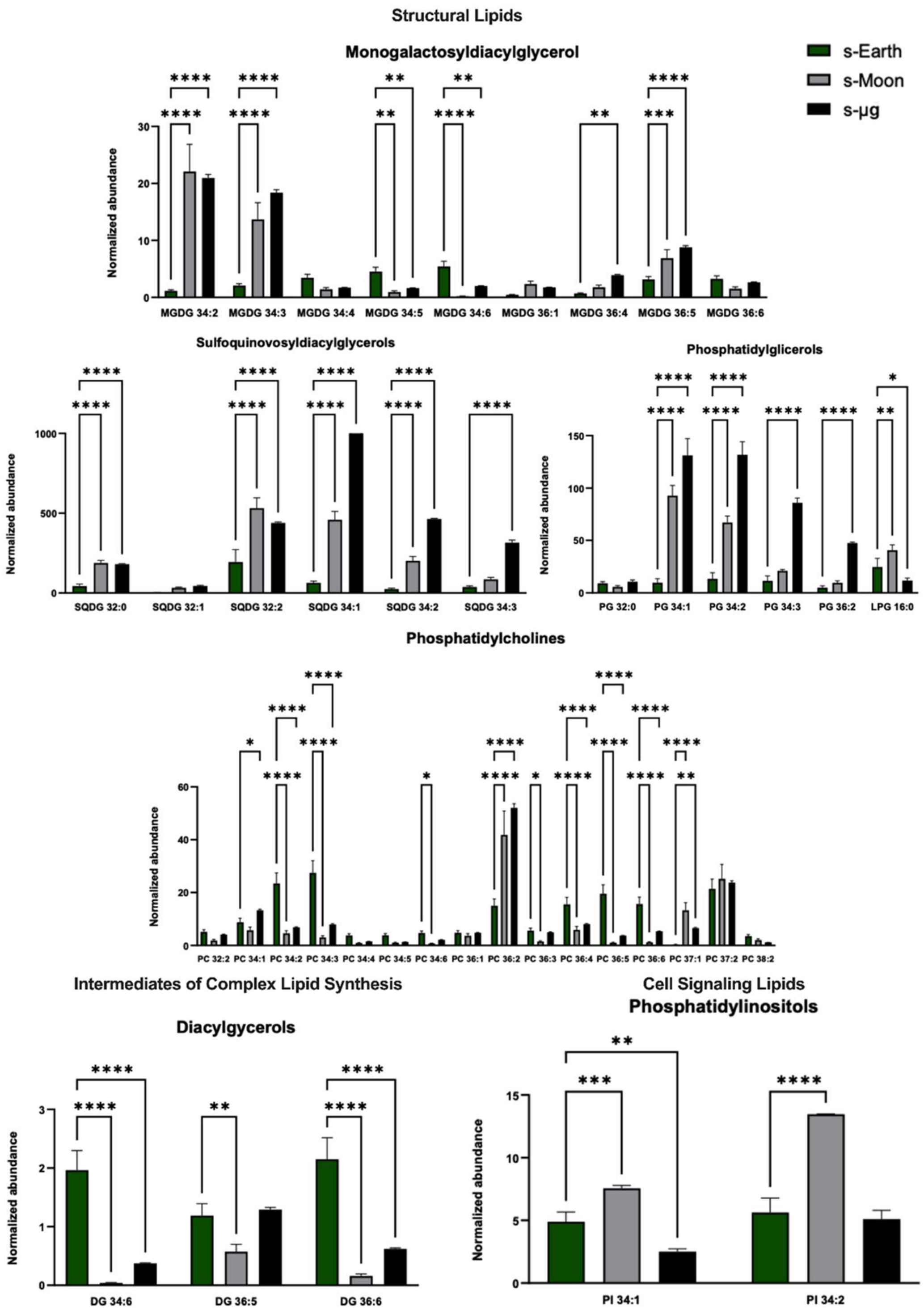

Supplementary Figure 2. Characterization of key lipid functional classes obtained from the separation of *Chlorella vulgaris* samples cultivated under simulated Earth (s-Earth), Moon (s-Moon), and microgravity (s-μg) conditions. Lipid classes are as follows: diacylglycerols (DG), digalactosyldiacylglycerols (DGDG), monogalactosyldiacylglycerols (MGDG), phosphatidylcholines (PC), triacylglycerols (TG), phosphatidylglycerols (PG), phosphatidylinositols (PI), and sulfoquinovosyldiacylglycerols (SQDG). Lipid functional classes are as follows: structural lipids as MGDG, SQDG, PG, and PC; intermediates of complex lipid synthesis as DG; cell signaling lipids as PI. Bars indicate mean ± SD (n = 3). Significant differences across gravity conditions are denoted by asterisks (*$p < 0.05$, **$p < 0.01$, ***$p < 0.001$, ** ** $p < 0.0001$).

Supplementary Tables

Supplementary Table 1. High resolution mass spectrometry characteristics of lipids identified in *Chlorella vulgaris* biomass. Lipid classes are as follows: diacylglycerols (DG), digalactosyldiacylglycerols (DGDG), monogalactosyldiacylglycerols (MGDG), phosphatidylcholines (PC), triacylglycerols (TG), phosphatidylglycerols (PG), phosphatidylinositols (PI), and sulfoquinovosyldiacylglycerols (SQDG).

Class of compounds	C#:sat#	Fatty Acids	Adduct	Formula	m/z		Error
					Theoretical	Detected	
	34:2	16:1/18:1	$[M+NH_4]^+$	$C_{43}H_{78}O_{10}$	772.5933	772.5932	-0.13
	34:3	16:1/18:2	$[M+NH_4]^+$	$C_{43}H_{76}O_{10}$	770.5777	770.5756	-2.73
	34:4	16:2/18:2	$[M+NH_4]^+$	$C_{43}H_{74}O_{10}$	768.5620	768.5606	-1.82
	34:5	16:2/18:3	$[M+NH_4]^+$	$C_{43}H_{72}O_{10}$	766.5464	766.5447	-2.22
**Monogalactosyldiacylglycerols**	34:6	16:3/18:3	$[M+NH_4]^+$	$C_{43}H_{70}O_{10}$	764.5307	764.5340	4.32
	36:1	18:0/18:1	$[M+NH_4]^+$	$C_{45}H_{84}O_{10}$	802.6403	802.634	-7.85
	36:4	18:1/18:3	$[M+NH_4]^+$	$C_{45}H_{78}O_{10}$	796.5933	796.5923	-1.26
	36:5	18:2/18:3	$[M+NH_4]^+$	$C_{45}H_{76}O_{10}$	794.5777	794.5764	-1.64
	36:6	18:3/18:3	$[M+NH_4]^+$	$C_{45}H_{74}O_{10}$	792.5620	792.5611	-1.14
	34:2	16:0/18:2	$[M+NH_4]^+$	$C_{49}H_{88}O_{15}$	934.6461	934.6420	-4.39
	34:4	16:2/18:2	$[M+NH_4]^+$	$C_{49}H_{84}O_{15}$	930.6148	930.6137	-1.18
**Digalactosyldiacylglycerols**	34:5	16:2/18:3	$[M+NH_4]^+$	$C_{49}H_{82}O_{15}$	928.5992	928.5977	-1.62
	34:6	16:3/18:3	$[M+NH_4]^+$	$C_{49}H_{80}O_{15}$	926.5835	926.5824	-1.19
	36:6	18:3/18:3	$[M+NH_4]^+$	$C_{51}H_{84}O_{15}$	954.6148	954.6132	-1.68
	32:0	16:0/16:0	$[M-H]^-$	$C_{41}H_{78}O_{12}S$	793.5141	793.5146	0.63
	32:1	16:0/16:1	$[M-H]^-$	$C_{41}H_{76}O_{12}S$	791.4985	791.4980	-0.63
**Sulfoquinovosyldiacylglycerols**	32:2	16:0/16:2	$[M-H]^-$	$C_{41}H_{74}O_{12}S$	789.4828	789.4832	0.51
	34:1	16:0/18:1	$[M-H]^-$	$C_{43}H_{80}O_{12}S$	819.5298	819.5269	-3.54
	34:2	16:0/18:2	$[M-H]^-$	$C_{43}H_{78}O_{12}S$	817.5141	817.5157	1.96
	34:3	18:3/16:0	$[M-H]^-$	$C_{43}H_{76}O_{12}S$	815.4985	815.4999	1.72
	32:0	16:0/16:0	$[M-H]^-$	$C_{38}H_{75}O_{10}P$	721.5025	721.5041	2.22
	34:1	16:0/18:1	$[M-H]^-$	$C_{40}H_{77}O_{10}P$	747.5182	747.5199	2.27
**Phosphatidylglycerols and lysophosphatidylglycerol**	34:2	16:0/18:2	$[M-H]^-$	$C_{40}H_{75}O_{10}P$	745.5025	745.5041	2.15
	34:3	16:0/18:3	$[M-H]^-$	$C_{40}H_{73}O_{10}P$	743.4869	743.4884	2.02
	36:2	18:1/18:1	$[M-H]^-$	$C_{42}H_{79}O_{10}P$	773.5338	773.5353	1.94
	16:0	16:0	$[M-H]^-$	C22H45O9P	483.2728	483.2801	2.69
**Phosphatidylinositols**	34:1	16:0/18:1	$[M-H]^-$	$C_{43}H_{81}O_{13}P$	835.5342	835.5350	0.96
	34:2	16:0/18:2	$[M-H]^-$	$C_{43}H_{79}O_{13}P$	833.5186	833.5206	2.40
	32:2	not determined	$[M+H]^+$	$C_{40}H_{76}NO_8P$	730.5381	730.5366	-2.1
	34:1	not determined	$[M+H]^+$	$C_{42}H_{82}NO_8P$	760.5851	760.5837	-1.8
	34:2	not determined	$[M+H]^+$	$C_{42}H_{80}NO_8P$	758.5694	758.5681	-1.7
	34:3	not determined	$[M+H]^+$	$C_{42}H_{78}NO_8P$	756.5538	756.5531	-0.9
	34:4	not determined	$[M+H]^+$	$C_{42}H_{76}NO_8P$	754.5381	754.5386	0.7
	34:5	not determined	$[M+H]^+$	$C_{42}H_{74}NO_8P$	752.5225	752.5212	-1.7
	34:6	not determined	$[M+H]^+$	$C_{42}H_{72}NO_8P$	750.5068	750.5061	-0.9
**Phosphatidylcholines**	36:1	not determined	$[M+H]^+$	$C_{44}H_{86}NO_8P$	788.6164	788.6161	-0.4
	36:2	not determined	$[M+H]^+$	$C_{44}H_{84}NO_8P$	786.6007	786.5991	-2.0
	36:3	not determined	$[M+H]^+$	$C_{44}H_{82}NO_8P$	784.5851	784.5841	-1.3
	36:4	not determined	$[M+H]^+$	$C_{44}H_{80}NO_8P$	782.5694	782.5683	-1.4
	36:5	not determined	$[M+H]^+$	$C_{44}H_{78}NO_8P$	780.5538	780.5530	-1.0
	36:6	not determined	$[M+H]^+$	$C_{44}H_{76}NO_8P$	778.5381	778.5372	-1.2
	37:1	not determined	$[M+H]^+$	$C_{45}H_{88}NO_8P$	802.6320	802.6340	2.5
	37:2	not determined	$[M+H]^+$	$C_{45}H_{86}NO_8P$	800.6164	800.6176	1.5
	38:2	not determined	$[M+H]^+$	$C_{46}H_{88}NO_8P$	814.6320	814.6329	1.1
	16:0	16:0	$[M-H]^-$	$C_{16}H_{32}O_2$	255.2330	255.2331	0.39
	16:1	16:1	$[M-H]^-$	$C_{16}H_{30}O_2$	253.2173	253.2181	3.16
	16:2	16:2	$[M-H]^-$	$C_{16}H_{28}O_2$	251.2017	251.2024	2.79
	17:0	17:0	$[M-H]^-$	$C_{17}H_{34}O_2$	269.2486	269.2492	2.23
	17:1	17:1	$[M-H]^-$	$C_{17}H_{32}O_2$	267.2330	267.2338	2.99
	18:0	18:0	$[M-H]^-$	$C_{18}H_{36}O_2$	283.2643	283.2653	3.53
	18:1	18:1	$[M-H]^-$	$C_{18}H_{34}O_2$	281.2486	281.2497	3.91
	18:2	18:2	$[M-H]^-$	$C_{18}H_{32}O_2$	279.2330	279.2342	4.30
	18:3	18:3	$[M-H]^-$	$C_{18}H_{30}O_2$	277.2173	277.218	2.53
	20:3	20:3	$[M-H]^-$	$C_{20}H_{34}O_2$	305.2486	305.2484	-0.74
	20:5	20:5	$[M-H]^-$	$C_{20}H_{30}O_2$	301.2173	301.2174	0.47
	22:0	22:0	$[M-H]^-$	$C_{22}H_{44}O_2$	339.3269	339.3266	-0.80

Class of compounds	C#:sat#	Fatty Acids	Adduct	Formula	m/z		Error
					Theoretical	Detected	
Diacylglycerols	34:6	16:3/18:3	$[M+NH_4]^+$	$C_{37}H_{60}O_5$	602.4779	602.4772	-1.16
	36:5	18:2/18:3	$[M+NH_4]^+$	$C_{39}H_{66}O_5$	632.5249	632.5242	-1.11
	36:6	18:3/18:3	$[M+NH_4]^+$	$C_{39}H_{64}O_5$	630.5092	630.5081	-1.74
Triacylglycerols	45:0	14:0/15:0/16:0	$[M+NH_4]^+$	$C_{48}H_{92}O_6$	782.7232	782.7229	-0.38
	46:0	14:0/16:0/16:0	$[M+NH_4]^+$	$C_{49}H_{94}O_6$	796.7389	796.7382	-0.88
	46:1	14:0/16:0/16:1	$[M+NH_4]^+$	$C_{49}H_{92}O_6$	794.7232	794.7219	-1.64
	47:0	15:0/16:0/16:0	$[M+NH_4]^+$	$C_{50}H_{96}O_6$	810.7545	810.7538	-0.86
	48:0	16:0/16:0/16:0	$[M+NH_4]^+$	$C_{51}H_{98}O_6$	824.7702	824.7697	-0.61
	48:1	16:0/16:0/16:1	$[M+NH_4]^+$	$C_{51}H_{96}O_6$	822.7545	822.7542	-0.36
	48:2	16:0/16:1/16:1	$[M+NH_4]^+$	$C_{51}H_{94}O_6$	820.7389	820.7378	-1.34
	48:3	16:0/16:0/16:3	$[M+NH_4]^+$	$C_{51}H_{92}O_6$	818.7232	818.7219	-1.59
	49:0	16:0/16:0/17:0	$[M+NH_4]^+$	$C_{52}H_{100}O_6$	838.7858	838.7851	-0.83
	49:1	15:0/16:0/18:1	$[M+NH_4]^+$	$C_{52}H_{98}O_6$	836.7702	836.7697	-0.60
	50:0	16:0/16:0/18:0	$[M+NH_4]^+$	$C_{53}H_{102}O_6$	852.8015	852.8007	-0.94
	50:1	16:0/16:0/18:1	$[M+NH_4]^+$	$C_{53}H_{100}O_6$	850.7858	850.7859	0.12
	50:2	16:0/16:0/18:2	$[M+NH_4]^+$	$C_{53}H_{98}O_6$	848.7702	848.7701	-0.12
	50:3	16:0/16:2/18:1	$[M+NH_4]^+$	$C_{53}H_{96}O_6$	846.7545	846.7544	-0.12
	50:4	16:0/16:3/18:1	$[M+NH_4]^+$	$C_{53}H_{94}O_6$	844.7389	844.7381	-0.95
	50:5	16:0/16:2/18:3	$[M+NH_4]^+$	$C_{53}H_{92}O_6$	842.7232	842.7222	-1.19
	50:6	16:0/16:3/18:3	$[M+NH_4]^+$	$C_{53}H_{90}O_6$	840.7076	840.7069	-0.83
	51:1	16:0/17:0/18:1	$[M+NH_4]^+$	$C_{54}H_{102}O_6$	864.8015	864.8009	-0.69
	51:2	16:0/17:1/18:1	$[M+NH_4]^+$	$C_{54}H_{100}O_6$	862.7858	862.7852	-0.70
	51:3	16:0/17:1/18:2	$[M+NH_4]^+$	$C_{54}H_{98}O_6$	860.7702	860.7694	-0.93
	51:4	16:0/17:1/18:3	$[M+NH_4]^+$	$C_{54}H_{96}O_6$	858.7545	858.7531	-1.63
	52:0	16:0/18:0/18:0	$[M+NH_4]^+$	$C_{55}H_{106}O_6$	880.8328	880.8324	-0.45
	52:1	16:0/18:0/18:1	$[M+NH_4]^+$	$C_{55}H_{104}O_6$	878.8171	878.8164	-0.80
	52:2	16:0/18:1/18:1	$[M+NH_4]^+$	$C_{55}H_{102}O_6$	876.8015	876.8012	-0.34
	52:3	16:0/18:1/18:2	$[M+NH_4]^+$	$C_{55}H_{100}O_6$	874.7858	874.7856	-0.23
	52:4	16:0/18:1/18:3	$[M+NH_4]^+$	$C_{55}H_{98}O_6$	872.7702	872.7699	-0.34
	52:5	16:0/18:2/18:3	$[M+NH_4]^+$	$C_{55}H_{96}O_6$	870.7545	870.7545	0.00
	53:2	17:0/18:1/18:1	$[M+NH_4]^+$	$C_{56}H_{104}O_6$	890.8171	890.8162	-1.01
	53:3	17:1/18:1/18:1	$[M+NH_4]^+$	$C_{56}H_{102}O_6$	888.8015	888.8004	-1.24
	54:0	18:0/18:0/18:0	$[M+NH_4]^+$	$C_{57}H_{110}O_6$	908.8641	908.8633	-0.88
	54:2	18:0/18:1/18:1	$[M+NH_4]^+$	$C_{57}H_{106}O_6$	904.8328	904.8321	-0.77
	54:3	18:1/18:1/18:1	$[M+NH_4]^+$	$C_{57}H_{104}O_6$	902.8171	902.8166	-0.55
	54:4	18:1/18:1/18:2	$[M+NH_4]^+$	$C_{57}H_{102}O_6$	900.8015	900.8011	-0.44
	54:5	18:1/18:1/18:3	$[M+NH_4]^+$	$C_{57}H_{100}O_6$	898.7858	898.7855	-0.33
	56:2	18:1/18:1/20:0	$[M+NH_4]^+$	$C_{59}H_{110}O_6$	932.8641	932.8642	0.11

Supplementary Table 2. Carotenoid/Chlorophyll (Car/Chl) ratios of *Chlorella vulgaris* samples cultivated under simulated Earth (s-Earth), Moon (s-Moon), and microgravity (s-μg) conditions. Data were reported as a mean ± SD (n = 3).

	**Carotenoids [μg g^{-1}]**	**Chlorophylls [μg g^{-1}]**	**Car/Chl ratio [\ ]**
**s-Earth**	1223.46 ± 503.22	631.8 ± 199.62	0.53 ± 0.06
**s-Moon**	2703.57 ± 425.15	1783 ± 167.1	0.66 ± 0.05
**s-μg**	3316.82 ± 89.08	2052.76 ± 11.76	0.62 ± 0.01

Supplementary Table 3. Unsaturation Index (UI) for each lipid head group class of *Chlorella vulgaris* samples cultivated under simulated Earth (s-Earth), Moon (s-Moon), and microgravity (s-μg) conditions. UIs were reported as a mean ± SD (n = 3) and significant differences between groups were reported in terms of *p*-values.

**Lipid class**	**s-Earth**	**s-Moon**	**s-μg**	**s-Earth vs. s-Moon**	**s-Earth vs. s-μg**	**s-Moon vs. s-μg**
MGDG	58 ± 10	75 ± 16	100 ± 3	-	-	-
DGDG	17.5 ± 3.0	18 ± 4	28 ± 1	-	-	-
SQDG	73 ± 36	527 ± 131	1533 ± 19	< 0.0001	< 0.0001	< 0.0001
PG	40 ± 16	154 ± 15	374 ± 28	< 0.005	< 0.0001	< 0.0001
PI	8.6 ± 3.6	17.2 ± 0.1	6.3 ± 0.8	-	-	-
PC	283 ± 48	118 ± 26	174 ± 5	< 0.00001	< 0.0005	-
DG	15 ± 3	2.0 ± 0.4	6.2 ± 0.2	-	-	-
TG	5.5 ± 0.9	15.0 ± 3.2	13.0 ± 0.4	-	-	-
